# Effectiveness and implementation of interventions for health promotion in urgent and emergency care settings: an umbrella review

**DOI:** 10.1186/s12873-023-00798-7

**Published:** 2023-04-06

**Authors:** Emma J. Adams, Lucy Morris, Goolnora Marshall, Frank Coffey, Philip D. Miller, Holly Blake

**Affiliations:** 1 School of Health Sciences, University of Nottingham, Nottingham, UK; 2 DREEAM: Department of Research and Education in Emergency Medicine, Acute Medicine and Major Trauma, Nottingham University Hospitals NHS Trust, Nottingham, UK; 3 East Midlands Academic Health Science Network, Nottingham, UK; 4 NIHR Nottingham Biomedical Research Centre, Nottingham, UK

**Keywords:** Emergency medicine, Urgent care, Health promotion, Intervention, Lifestyle, Review

## Abstract

**Background:**

Urgent and emergency care (UEC) settings provide an opportunity to prevent ill-health and promote healthy lifestyles with potential to screen and deliver interventions to under-served, at-risk populations. The aim of this study was to synthesise and summarise the evidence on the effectiveness and implementation of interventions for health promotion in UEC settings.

**Methods:**

PubMed and Embase (OVID) databases were used to search for studies published in English between January 2010 and January 2023. Systematic reviews and meta-analyses of studies that examined the effectiveness or implementation of face-to-face health promotion interventions for lifestyle behaviours delivered in UEC settings were eligible. Extracted data were synthesised and qualitatively summarised by lifestyle behaviour. Reviews were quality assessed using AMSTAR 2.

**Results:**

Eighteen reviews met the inclusion criteria; all included studies were conducted in emergency departments or trauma units. We identified 15 reviews on alcohol interventions (13 on effectiveness; 2 on implementation) and 3 on smoking interventions (effectiveness). There were no reviews of intervention studies targeting physical activity or diet and nutrition. There was heterogeneity across studies for study design, target populations, intervention design and content, comparator/control groups and outcomes assessed. The effectiveness of alcohol and smoking interventions in UEC settings varied but some reviews provided evidence of a significant decrease in alcohol consumption, alcohol-related outcomes and smoking in intervention groups, particularly in the short-term and in specific population groups. Research has focused on ‘brief’ interventions as part of screening, brief intervention and referral to treatment (SBIRT) approaches. Interventions are delivered by a wide range of staff with substantial variation in design. Alcohol brief interventions appear to be acceptable to UEC patients but clinicians face barriers in delivering them.

**Conclusions:**

UEC settings have been under-researched and appear to be under-utilised for delivering health promotion activities, except for alcohol prevention. Review level evidence suggests alcohol and smoking interventions are warranted in some population groups. However, further research is needed to determine the optimal intervention design, content and delivery mode for lifestyle behaviours which are suitable for implementation in UEC settings and promote long-term intervention effectiveness. Changes in clinical practice may be needed, including increased training, integration into service delivery and supportive policy, to facilitate the implementation of SBIRT for lifestyle behaviours. Interventions may need to be delivered in the wider UEC system such as urgent care centres, minor injury units and walk-in centres, in addition to emergency departments and trauma units, to support and increase health promotion activities in UEC settings.

**Supplementary Information:**

The online version contains supplementary material available at 10.1186/s12873-023-00798-7.

## Background

Urgent and emergency care (UEC) settings (such as emergency departments (ED), trauma units, urgent care centres, minor injury units and walk-in centres) have an important role to play in identifying at-risk individuals and delivering health promotion activities to address unhealthy lifestyle behaviours and prevent and manage non-communicable disease. UEC settings are attended by large numbers of patients, some of whom are from lower socio-economic backgrounds and may have less access to primary care, such as General Practitioners (GPs) and preventative care facilities [1]. These settings enable at-risk patients to be reached, are seen as a credible source of information, allow for screening and brief conversations about lifestyle behaviours, and provide the opportunity for a ‘teachable moment’ when patients may be receptive to advice from a healthcare professional related to the reason for their admission [2]. There are potential benefits for patients, health services and society for using UEC settings to deliver health promotion interventions. These include prevention of ill-health, improved health and improved quality of life for patients; reduced health inequalities; reduced healthcare costs; and benefits for the economy and employers due to reductions in days lost at work and costs from sickness absence [3, 4].

Brief interventions have been widely researched in primary care health settings for addressing lifestyle behaviours such as alcohol consumption, smoking, physical activity and diet and nutrition/weight management [5–8]. The process is often referred to as screening and brief intervention (SBI) and sometimes includes referral to treatment (SBIRT). Screening refers to the rapid assessment of a patient’s current behaviour and ideally identification of the advice or treatment that might be needed to help them. Brief interventions, sometimes called ‘brief advice’, are short, one-off, structured conversations about a lifestyle behaviour varying in length from 5 to 60 min. They aim to motivate and support individuals to consider changing their behaviour and may include motivational interviewing (a communication approach to help people make attitudinal or behavioural change) [9], or may be supplemented with additional materials (e.g., a patient leaflet with information and resources, or information about local support services). Referral to treatment is used when a patient requires additional support or more extensive and longer-term interventions provided by other hospital departments, GPs or local services and support groups. There is increasing interest in the use of SBIRT in UEC settings as an approach to change health behaviours and improve health in patients presenting with chronic illnesses or injuries directly or indirectly related to unhealthy lifestyles.

Despite their potential, UEC settings appear to have been underutilised for health promotion. Except for alcohol interventions, there appears to be limited research on the implementation and effectiveness of health promotion activities in this setting. Whilst a review and clinical guidelines exist for interventions to tackle alcohol consumption in healthcare settings [5, 10], these have often been reported under the umbrella of, or combined with, primary care. As a result, there may be a lack of awareness of these guidelines amongst healthcare professionals working in UEC settings. UEC should be considered as a unique setting as the quality and outcome of interventions in different settings can vary [10]. Setting-specific guidance and policy should be provided to increase awareness of the importance and profile of health promotion activities among UEC staff.

Whilst early work was undertaken to review the literature and establish a framework for health promoting emergency departments [2, 11], there appears to have been little published on the wider use of UEC settings for health promotion in the last twenty years. An initial literature search conducted by the authors found no recent overview of the evidence for the effectiveness and implementation of brief interventions for health promotion in UEC settings has been published. An up-to-date summary of the evidence is required by healthcare professionals, managers and decision-makers to inform the development and improvement of lifestyle health promotion interventions and services in UEC settings in clinical practice. One such strategy is to undertake an umbrella review to summarise the review level evidence on the topic [12]. The aim of this umbrella review was to synthesise and summarise the evidence on the effectiveness and implementation of interventions for health promotion for lifestyle behaviours (alcohol consumption, smoking, physical activity, diet and nutrition) in UEC settings and outline the implications for future research and clinical practice.

## Methods

A rapid umbrella review was undertaken due to the need for evidence to be synthesised quickly to inform service improvements [13–15]. A protocol was developed and agreed with the study team before commencing the review. The Preferred Reporting Items for Systematic Reviews and Meta-Analyses (PRISMA) Guidelines 2020 were used to conduct and report this study [16].

### Search strategy

Searches were carried out using two databases, PubMed and Embase (Ovid), on 24^th^ October 2021. The search was repeated on 17^th^ January 2023 to identify any additional papers published since the original search was undertaken. Search terms were identified by the study team and MeSH descriptors were checked in PubMed to identify any additional terms; combinations of these sets of terms were used to identify articles (Additional File 1).

### Inclusion and exclusion criteria

Articles were included that met the following criteria: 1) *Population*: intervention targeted any patient aged 11 years or over attending UEC for any reason; or those involved with delivering such interventions e.g., clinicians; 2) *Intervention:* included any form of one-to-one and face-to-face intervention, or the implementation of such interventions, which aimed to improve health or prevent ill-health by changing patient behaviour related to alcohol consumption/misuse, smoking, physical activity or diet and nutrition (including weight management or obesity) and was delivered in a UEC setting; 3) *Comparator:* included any or no comparator/control conditions; and 4) *Outcomes*: any outcome related to the effectiveness of brief interventions and/or the cost-effectiveness of brief interventions and/or implementation outcomes related to brief interventions, including but not limited to who delivered the intervention, staff training, delivery mode, timing, frequency and duration, content, barriers and facilitators, staff and patient views; 5) articles were systematic reviews or meta-analyses; and 6) articles were written in English and were published between 1^st^ January 2010 and 17^th^ January 2023.

Articles were excluded if they met the following criteria: 1) included interventions delivered to groups, or solely online or by telephone; 2) focused on non-health promotion related interventions in UEC settings, for example therapeutic or treatment-based interventions; 3) included interventions delivered in a non-urgent or non-emergency healthcare setting e.g., in primary care, at a planned general practitioner, nurse or hospital appointment, or during in-patient hospitalisation, where the findings from these could not be separated from UEC interventions; 4) articles were narrative reviews, scoping reviews, abstracts only, clinical guidelines, opinion pieces, magazine and newspaper articles, case reports and conference proceedings.

### Study selection

**Fig. 1:**
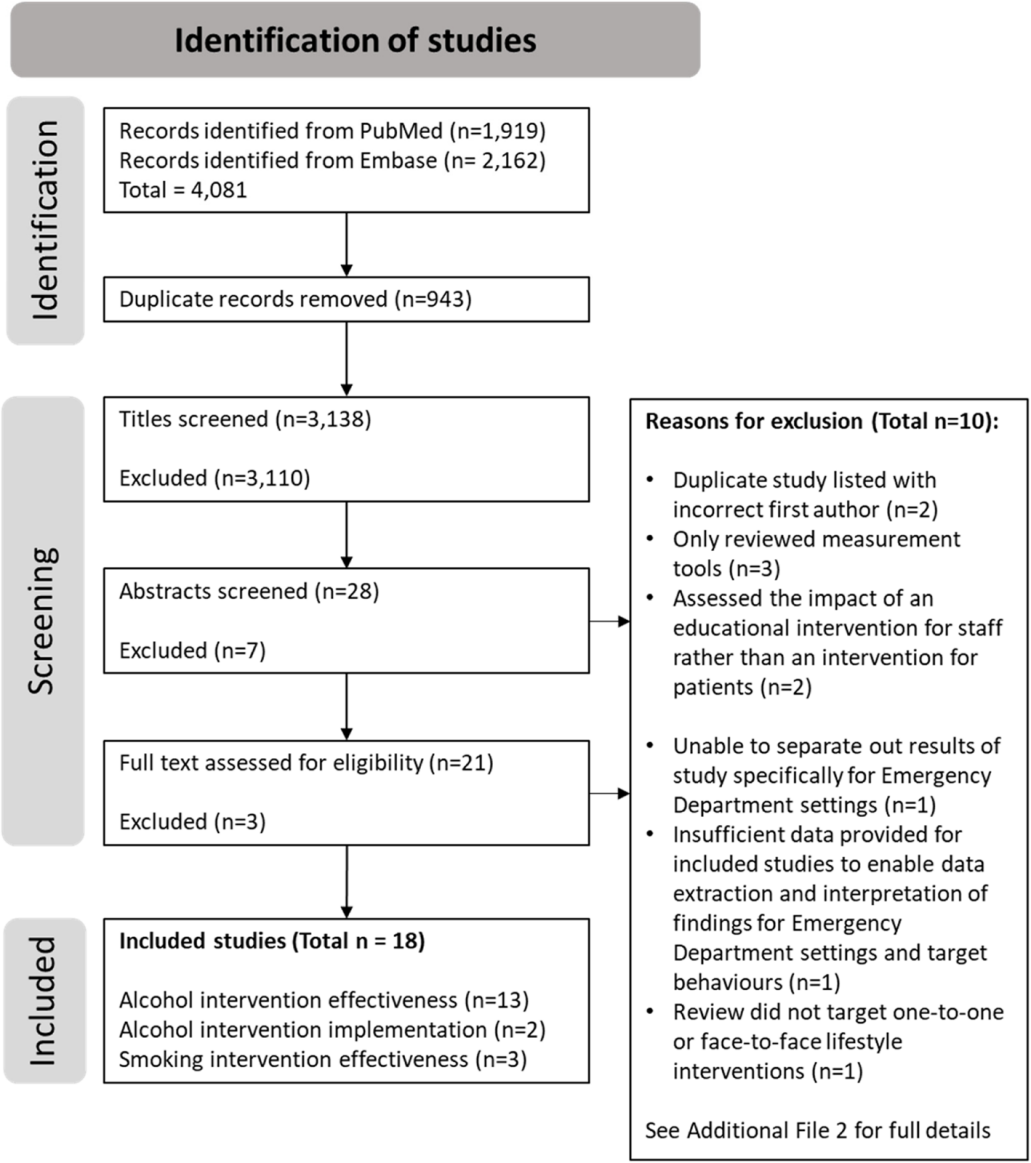
Literature search flowchart

The titles of articles were screened and assessed by one reviewer (EA). For any publications where it was unclear whether they met the inclusion/exclusion criteria, the abstract was reviewed. Articles which appeared to meet the inclusion/exclusion criteria were sourced and the full text assessed. Any uncertainties regarding the inclusion or exclusion of a study were discussed with the study team. In addition, the titles and abstracts of 10% of included articles were reviewed for inclusion by an independent researcher; and any discrepancies were resolved by discussion. Further details of the number of included/excluded reviews at each stage are provided in Fig. 1.

### Data extraction

A data extraction table was used to collect key information from each review. Data were extracted by one reviewer (EA). Additional reviewers (LM, GM) independently extracted data for 20% of included studies to check for accuracy, and any discrepancies were resolved by discussion. Key data extracted included: authors and year of publication; review aims (topic); target lifestyle risk factor/behaviour(s); review inclusion criteria (including study population (P); intervention description (I); comparator/control conditions (C); outcomes (O); the number of included studies by study design and country; number of participants and participant characteristics; outcomes assessed; screening tools used; intervention content and control/comparator conditions; and findings related to intervention effectiveness, cost-effectiveness and/or implementation outcomes. Where no summary data were provided in the text, data were extracted from the information provided in tables or figures across the included studies where available.

### Quality assessment

A quality assessment of included reviews was undertaken by one reviewer (EA) using AMSTAR 2 [17]*.* AMSTAR 2 is a reliable and valid measurement tool to assess the methodological quality of systematic reviews which include randomised and non-randomised clinical trials.

### Synthesis, summarising and reporting of results

The findings from this systematic review of reviews were collated by lifestyle behaviour and whether the articles assessed effectiveness or implementation. In addition, effectiveness studies which included information about implementation (e.g., who delivered the intervention) were included in the summary of findings for implementation studies. Due to the heterogeneity of the study and intervention designs, a qualitative synthesis was undertaken to summarise the extracted data for systematic reviews and quantitative findings for meta-analysis were summarised. Future research needs are identified along with the implications of the findings for clinical practice.

## Results

### Results of search strategy

The systematic literature search identified 3,138 reviews that were potentially relevant for inclusion in this umbrella review. After reviewing titles, abstracts, and the full text of papers, eighteen studies were included in this review (Fig. 1.). Details of excluded papers which underwent abstract or full text review are included in Additional File 2.

### Review characteristics

All studies included in the reviews took place in emergency departments or trauma centres (data not shown). The majority of reviews focused on alcohol consumption (15 reviews; 13 on the effectiveness of interventions [18–30]; and two implementation-related studies [31, 32]) with a further three reviews for smoking (related to intervention effectiveness [33–35]). There were no published reviews of brief interventions in UEC settings for physical activity or diet and nutrition. The characteristics of the 18 included reviews are summarised in Table 1. Twelve studies were systematic reviews (11 alcohol [18, 19, 22, 24–26, 28–32]; one smoking [34]) and six studies were systematic reviews and meta-analyses (four alcohol [20, 21, 23, 27]; two smoking [33, 35]). Ten reviews included randomised controlled trials (RCTs) only (eight alcohol [18, 20, 21, 23, 26, 27, 30, 32]); two smoking [33, 35]) and eight reviews included a wide range of study designs including RCTs and observational studies, prospective studies, non-randomised studies, pre/post only studies, quasi-randomised studied, qualitative studies and practice-based evidence (seven alcohol [19, 22, 24, 25, 28, 29, 31]; one smoking: [34]). Two reviews considered only studies from the United States of America (USA) (alcohol [22, 30]). The remainder of reviews included studies from a wide range of countries including the USA, Australia, Brazil, and from across Europe including the United Kingdom, Germany, Switzerland, Sweden, Poland and Spain, although the majority of studies were undertaken in the USA. Six reviews targeted all age groups (three alcohol intervention reviews [18, 25, 27] and three smoking intervention reviews [33–35]); five alcohol intervention reviews [20–22, 24, 28] and one alcohol implementation review [32] targeted adults only; and five alcohol intervention reviews targeted children, adolescents or young people only (age range 11–25) [19, 23, 26, 29, 30]. One alcohol implementation review included clinician-reported barriers rather than patient outcomes [31].

**Table 1:** Characteristics of the included reviews

Author (year)	Type of review	Search period	Topic	Population target age	Types / number of studies included	Countries	Sample size
**Effectiveness studies (Alcohol)**							
Barata et al., (2017) [18]	Systematic review	Jan 1966 to April 2016	Effectiveness of ED brief interventions for patients at risk for alcohol use disorder (AUD) for reducing alcohol intake and preventing alcohol-related injuries	All ages	RCTs (35 studies)	USA (33 studies); UK (1 study); Australia (1 study)	No data
Diestelkamp et al., (2016) [19]	Systematic review	Not limited to a specific range of years	BIs targeting adolescents and young adults following an alcohol-related event including non-Anglo-Saxon evidence and parameters indicative of acceptance, participation and implementation	Aged between 12 and 25 years	RCTs (7 studies) Additional evidence: Practice projects (6 studies) Non-randomised pilot study (1 study) Observational study (1 study)	*RCTs:* USA (4 studies); Australia (1 study); Brazil (1 study); Germany (1 study) *Additional evidence:* Germany (5 studies); Australia (2 studies); Switzerland (1)	1,125 participants, ranging from a minimum of 94 to a maximum of 254 participants
Elzerbi et al., (2015) [20]	Systematic review and meta-analysis	January 2007 to August 2014	Efficacy of BI at 6- and 12-month follow-up in primary health care and emergency department (ED) studies; examine variance in study outcome by the geographical region (European versus non-European)	Aged 18–64 years old	RCTs (8 studies) (RCTs in Primary Health Care (20 studies)—excluded from this review)	*Europe (4 studies):* UK (2 studies); Poland (1 study); Switzerland (1 study) *Non-Europe:* USA (4 studies)	Total 4,799 participants: European = 2,465 Non-European = 2,334
Elzerbi et al., (2017) [21]	Systematic review and meta-analysis	Published before September 2016	Direction and magnitude of difference between BI versus control group for reducing alcohol consumption in targeted injury and non-injury-specific studies at standard trial follow-up points	Aged 16–64 years old	RCTs (23 studies)	USA (15 studies); UK (2 studies); Switzerland (2 studies); Australia (1 study); Sweden (1 study); Germany (1 study); Poland (1 study)	15,173 participants
Kodadek et al., (2020) [22]	Systematic review	Up to November 2018	Preventive efficacy of SBIRT in adult patients treated for injury in the ED, trauma centre, or hospital setting	Adult patients	Total (11 studies): RCTs (5 studies) Observational studies (6 studies): Retrospective cohort (1 study) Prospective (1 study) Non-randomised (4 studies)	USA studies only	3,119 patients comprised of 1,897 patients who received SBIRT and 1,222 patients who received usual trauma care
Kohler & Hofmann (2015) [23]	Systematic review and meta-analysis	Up to 24th September 2013	Changes in alcohol consumption after brief motivational interventions for young people with existing alcohol use problems, who were admitted to an emergency care unit alcohol positive, with an alcohol-related trauma, or with a history of elevated alcohol consumption	Included some young people ≤ 18 years; excluded > 25 years	RCTs (6 studies)	USA (5 studies); Brazil (1 study)	1,433 participants included in the meta-analysis. Sample size varied from 94 to 567 participants
Landy et al., (2016) [24]	Systematic review	Published in June 2014 and earlier	Effectiveness of BIs for alcohol misuse in EDs	Adult sample (majority 18 to 65 years of age)	Total (33 studies): RCTs (18 studies) Pre/post designs (14 studies) Secondary analysis (1 study)	USA (16 studies); UK (6 studies); Sweden (4 studies); Switzerland (3 studies); Australia (1 study); Germany (1 study); Poland (1 study); Spain (1 study)	No data
McGinnes et al., (2016) [25]	Systematic review	1996 to 19th January 2015	Effectiveness of preventive health interventions in the ED setting of 10 min or less or involving technology in reducing harmful or risky drinking; characteristics of effective interventions and feasibility issues or barriers to the introduction of preventive health interventions in EDs	Adults and adolescents	Total (13 studies): RCTs (12 studies) Quasi-randomised trial (1 study)	USA (8 studies); Australia (2 studies); Germany (1 study) Sweden (1 study); UK (1 study)	No data
Newton et al., (2013) [26]	Systematic review	1985 to April 2011	Effect of ED-based BI compared with standard or other care in reducing harmful and hazardous alcohol and other drug use and associated morbidities in youth	Patients predominantly ≤ 19 years	RCT s (9 studies)	USA (8 studies); Australia (1 study)	No data
Schmidt et al., (2016) [27]	Systematic review and meta-analysis	January 2002 to August 2015	Impact of BI in ED on alcohol consumption	All ages	RCTs (including cluster randomisation or randomisation by time sequence) (28 studies from 33 publications)	USA (22 studies); UK (4 studies); Poland (2 studies); Australia (1 study); Brazil (1 study); Germany (1 study); Sweden (1 study); Switzerland (1 study)	14,456 patients
Simioni et al., (2015) [28]	Systematic review	Up to 31st December 2013	Interventions that effectively increase alcohol treatment utilization among ED patients with AUDs	Adults 18 or older	Total (7 studies): RCTs (4 studies) Controlled Clinical Trials (2 studies) Non-randomised controlled trial (1 studies)	USA (5 studies); UK (1 study); France (1 study)	No data
Taggart et al., (2013) [29]	Systematic review	1990 to April 2012	Use of brief ED interventions in the college-age population	18–20 year olds	Total (7 studies): Prospective randomized trials (6 studies); Prospective (1 study)	USA (5 studies); Brazil (1 study); Australia (1 study);	No data
Yuma-Guerrero et al., (2012) [30]	Systematic review	Published before January 2011	SBIRT with adolescent patients in acute care settings	Adolescents (11–21 years of age)	RCTs (7 studies)	USA studies only	No data
**Effectiveness studies (Smoking)**							
Lemhoefer et al., (2017) [33]	Systematic review and meta-analysis	4th October 2010 to 15th May 2015	Update of the systematic review and meta-analysis of RCTs published in 2012 (Rabe et al., 2013)	Any age	RCTs (4 additional studies; one published as an abstract)	US (3 studies); Canada (1 study)	1,392 participants overall
Pelletier et al., (2014) [34]	Systematic review	Up to February 2014	Smoking cessation interventions for patients in the adult or paediatric emergency care including effectiveness, feasibility, and appropriateness assessed by their impact on smoking cessation, all-cause mortality, patient satisfaction, practitioner time spent, non-practitioner time spent, & cost per quit	Any age	13 studies with lowest risk of bias included: RCTs (12 studies); Prospective study (1 study)	USA (11 studies); Germany (1 study); Turkey (1 study)	No data
Rabe et al., (2013) [35]	Systematic review and meta-analysis	Up to 4^th^ October 2010	Efficacy of Emergency Department-initiated tobacco control (ETC)	Any age	RCTs (7 studies)	USA (6 studies); Germany (1 study)	1,986 participants overall (sample size range: 40 to 1,044 study participants)
**Implementation studies**							
Gargaritano et al., (2020) [31]	Systematic review	Up to 27^th^ June 2019	Clinician perceived barriers for the implementation of alcohol screening and brief intervention in hospital settings	Not applicable (Clinicians of any age)	Total (25 studies): Quantitative studies (16 studies); Qualitative studies (8 studies); Mixed methods (1 study)	USA (9 studies); Canada (2 studies); UK (2 studies); Spain (1 studies); Australia (6 studies); Denmark (3 studies); Tanzania (1 studies); Taiwan (1 studies)	No data
Pedersen et al., (2011) [32]	Systematic review	Up to 25^th^ November 2010	Acceptance of screening and intervention and adherence to intervention programmes among emergency department (ED) and surgical patients with AUDs	Adults	Total (33 studies): RCTs (31 studies); Conducted in ED (28 studies); Conducted in surgical patients (5 studies)	USA (16 studies); UK (6 studies); Spain (3 studies); Australia (2 studies); Sweden (2 studies); Denmark (1 study); Finland (1 study); Germany (1 study); Switzerland (1 study)	Total population given in 18 of 28 studies; median size was 5,640 ranging 697 to 32,965 patients

*AUD* Alcohol use disorder, *BI* Brief intervention, *COM-B* Capability, Opportunity, Motivation – Behaviour, *ED* Emergency Department, ETC Emergency Department-initiated tobacco control, *RCTs* Randomised control trials, *SBIRT* screening, brief intervention and referral to treatment

The full inclusion criteria for each effectiveness review in relation to study population (P); intervention description (I); comparator/ control conditions (C); and outcomes (O) are provided in Additional File 3. There was considerable heterogeneity across the reviews in each of these domains. *Study population:* Patients were included in studies for a variety of reasons. For example, they were admitted to UEC for any reason [28], following an alcohol-related event or injury [19], were intoxicated/tested positive for alcohol consumption [27], screened positive for alcohol misuse or hazardous or harmful drinking [20, 21, 24], had known alcohol use disorders or symptoms of an alcohol-related disorder [18, 25], or following trauma [22]. For smoking, any current smoker was included regardless of reason for admission. *Interventions:* Details provided about included interventions varied across reviews. Where criteria were specified, a broad range of interventions were included with varying definitions of what constituted a brief intervention regarding duration, number of sessions and content. For example, some reviews specified it as single session of five to 30 min [24], whereas others included multiple sessions of up to 60 min or booster sessions (either face-to-face or by telephone) [19, 27]. One study focused on motivational interviewing [23], another included ultra-brief interventions defined as any face-to-face intervention of less than 10 min or a non-face-to-face intervention involving technology [25]. Many reviews did not specify a clear definition of the content or specific criteria for the intervention designs to be included in the review, thus there was considerable heterogeneity in the interventions reported. *Comparators/control conditions:* most studies allowed no treatment, standard care, screening only, a brief intervention of a different intensity or an alternative intervention (e.g., leaflets or information booklet) as a comparator or control condition with a mixture of conditions being used across studies. Typically, there was no description of what standard care comprised. *Outcomes:* For alcohol, most reviews included a measure of alcohol consumption, such as quantity and/or frequency, however the exact measure and frequency of follow-up varied across studies. One review focused on treatment utilisation following an ED-based intervention rather than alcohol consumption or related outcomes [28]. Some studies assessed other outcomes related to alcohol such as injury or re-injury [19, 22, 24, 26]; healthcare use, ED admissions, readmissions or hospitalisations [24, 26]; alcohol-related risky behaviour such as driving offences or drink driving [18, 19, 22, 24, 26] or unprotected sex [24]; or referral to treatment [18, 19, 28]. For smoking, outcomes were self-reported seven days abstinence or smoking cessation rate [33–35].

Two studies specifically investigated implementation related outcomes; these were clinician barriers to delivering brief interventions [31] and adherence and acceptance [32]. A further eleven studies reported varying levels of information about the implementation of the interventions in addition to intervention effectiveness (alcohol [18–21, 23–28]; smoking [34]). Ten studies provided examples of personnel who delivered the interventions [18–21, 23–28]; and six studies considered other implementation outcomes such as training for intervention delivery staff [18, 23], participation rates [19], acceptance [19], implementation (including feasibility and stakeholder support) [19], follow-up or retention rates [25, 27] and intervention time taken [34].

### Quality assessment

Findings from the quality assessment are reported in Additional File 4. Based on the critical domains of the AMSTAR 2 assessment tool [17], quality varied across reviews with risk of bias satisfactorily assessed in eight out of the eighteen studies. Five out of the eighteen studies registered or used a previously published protocol. All studies searched at least two databases and provided the key words and search strategy used. Of the six studies which included a meta-analysis, four reviews included only low risk of bias studies in their analyses.

### Findings of the reviews

#### Interventions for alcohol

A wide variety of questionnaire-based screening tools were used across studies to assess different outcomes in different populations related to alcohol consumption, and alcohol-related negative consequences such as drink driving or other risky behaviours; some studies included assessments of blood alcohol concentration or alcohol in saliva or breath in addition to, or instead of, self-report measures (Additional File 5). Full details of the participant characteristics, intervention design and content, and comparator/control conditions are presented in Additional File 6. Participant characteristics were reported in seven studies but were limited to the age of participants [18, 23, 25–27, 30] and gender [19, 23, 25–27]. The ages and the gender ratio of participants varied across studies included in the reviews. There was considerable heterogeneity in intervention design and content for the intervention and control/comparator groups across studies included in the reviews. Interventions varied in duration (from less than 5 min to 60 min), content (varying levels of brief advice, counselling, or use of motivational interviewing; provision of generic or tailored information via different media such as verbally, printed materials such as booklets or via text message; differing number of sessions, or referral to counselling or booster sessions (by telephone or face-to-face). Control/comparator groups received standard care (not described), screening only, or in most studies provision of written or brief verbal information, text messages, referrals, or a follow-up phone call.

Findings for the effectiveness of interventions for reducing alcohol consumption outcomes were mixed (Table 2). Reductions in alcohol consumption (quantity or frequency) in both the intervention and comparator groups were reported in several reviews [18, 19, 22–24, 30]. Significant differences between intervention and comparator groups favouring the intervention group were reported in some studies [18, 19, 22, 24, 25, 27, 29, 30]. In meta-analyses, positive effects were reported on reductions in alcohol consumption at varying time points or for different alcohol consumption outcomes (6 and 12 months [20]; 6 months only [21]; and significant lower frequency (but not quantity) of drinking in MI groups, [23]). Effects tended to dissipate over time between 3-, 6- and 12-month follow-up assessments [18, 21, 23, 24, 27].

**Table 2:** Key findings for the effectiveness of interventions for alcohol consumption outcomes

Author (year)	Intervention	Outcome(s)	Number of studies	Results
Barata et al., (2017) [18]	SBIRT (no specified duration or sessions)	Reduction in number of drink days and number of units per drink day	16/35	• Reduction in both the control and intervention groups
			9/16	• Higher reduction in intervention group compared to control group
			13/35	• Significant differences between control and intervention groups
			17/35	• No intervention effect for reduction in alcohol consumption
Diestelkamp et al., (2016) [19]	BI (max 60 min); max 3 sessions; 1 in ED	Alcohol consumption	7/8	• Reductions in alcohol consumption regardless of form of care
			2/8	• Significant differences across conditions
			2/8	• Between-group differences in reductions of alcohol use for sub-groups only (those testing positive for referral to alcohol treatment at baseline; females)
Elzerbi et al., (2015) [20]	BI (max 30 min); max 4 sessions	Grams of alcohol consumed per week at 6 and 12 months	8	• Statistically significant difference in reducing grams of alcohol consumed per week between intervention and control groups at 6 and 12 months
Elzerbi et al., (2017) [21]	BI (max 45 min); max 4 sessions	Grams of alcohol consumed per week at less than or equal to 5, 6 and 12 months	23	• No effect at 6 or 12 months when combining injury and non-injury studies *Targeted injury studies:*
				• Small effect in favour of the intervention compared to the control at 6 months
				• No effect of the intervention compared to the control at 12 months*Non-injury-specific studies:*
				• Effect in favour of the intervention compared to the control at 6 and 12 months
Kodadek et al., (2020) [22]	SBIRT (no specified duration or sessions)	Alcohol consumption	2/7	• Statistically significant decrease in alcohol consumption following intervention
			1/7	• Non-statistically significant lower alcohol consumption in intervention group
			2/7	• Lower alcohol consumption in intervention and control groups
			2/7	• Decreased alcohol consumption in the intervention group, but no control group
Kohler & Hofmann (2015) [23]	Motivational interviewing (no specified duration or sessions)	Changes in alcohol consumption (drinking frequency or drinking quantity)		*Systematic review:*
			1/6	• No significant change in drinking frequency/quantity regardless of intervention used
			5/6	• Young people consumed significantly less alcohol regardless of use of MI
			3/6	• Lowest amount of drinking at 3- or 6-month follow-up; rising consumption post 6 months
				*Meta-analysis:*
			6	• MI reduced alcohol consumption at least as much as a control intervention
				• Frequency of drinking was significantly lower in the MI groups
				• MI showed no advantage over control interventions in reducing drinking quantity
				• MI is more efficacious than other interventions in reducing drinking frequency (USA studies only)
Landy et al., (2016) [24]	BI (5- 60 min); single session; no additional booster sessions	Alcohol consumption: reduction in number of units of alcohol per occasion (17 studies) / reduction in frequency of drinking/binge drinking (8 studies)		*At 3 months:*
			9	• All studies found a significant reduction in alcohol consumption
			2/9	• Significantly greater reduction in alcohol consumption in the BI group compared to control group
			3/9	• Significant reduction in alcohol consumption in the intervention and control groups, no significant differences between group
			1/9	• Significant reductions in alcohol consumption, but no control group
				*At 6 months:*
			3/17	• Significantly greater reduction in alcohol consumption in the BI group compared to control group
			3/17	• Significant reduction in alcohol consumption in the intervention and control groups, no significant differences between groups
			2/17	• Significant reductions in alcohol consumption in BI group, but no control/comparison group
			2/17	• No significant difference between the BI and control groups
			1/17	• No significant differences between three conditions (patient leaflet, brief advice (5 min) and brief lifestyle counselling (20 min)
			1/17	• Fewer participants in BI group met criteria for at-risk drinking comparted to control group
				*At 12 months:*
			4/14	• Significantly greater reduction in alcohol consumption in the BI group compared to control group
			7/14	• No significant difference between the BI and control groups
			1/14	• No differences between BI group and group receiving extended counselling
McGinnes et al., (2016) [25]	Ultra BI; (10 min or less)	Quantity of alcohol consumed Frequency of alcohol use Binge drinking	6/13	• Significant reduction in the quantity of alcohol consumed
			0/13	• No studies showed a reduction in frequency of alcohol use
			3/13	• Significant reduction in binge drinking
Newton et al., (2013) [26]	BI (time limited); 1 or 2 sessions	Frequency of alcohol use		*Targeted BIs:*
			1/9	• MI and standard care favoured at different time points, no statistically significant group differences
			1/9	• MI group greater reduction in alcohol use compared to comparison group, no statistically significant group differences
			1/9	• Youth attending post-ED community service after receiving BI or standard care reported a greater reduction in alcohol consumption compared to those who did not attend the post-ED community service
				*Universal BIs:*
			1/9	• MI, handouts and standard care favoured at different time points, no statistically significant group differences
			3/9	• Trends observed in high-risk groups (alcohol misuse at baseline) for reducing drinking frequency, high volume drinking days and maximum number of alcohol-based drinks per day
			1/9	• Effect on alcohol misuse and binge drinking in a subset of patients who reported previous drinking and driving behaviours
Schmidt et al., (2016) [27]	BI (5–40 min); 1 to 4 sessions	Quantity (alcohol consumption per week/month), intensity (alcohol consumption per day/occasion) and number of heavy drinking episodes at 3, 6 and 12 months	22	• Significant reduction in alcohol use (quantity) in the intervention group at 12 months
			14	• Significant reduction in alcohol use (intensity) in the intervention group, greatest at 3 month follow up
			18	• Slightly higher reductions in the number of heavy drinking episodes (binge drinking) in the intervention group at 6 and 12 months
Taggart et al., (2013) [29]	Standardised treatment to reduce alcohol intake	Changes in alcohol intake patterns	7/7	• All studies found reductions in alcohol intake patterns in the intervention group; some between-group differences were not statistically significant
Yuma-Guerrero et al., (2012) [30]	BI (no specified duration or sessions)	Alcohol consumption	6/7	• Positive alcohol consumption effects for all participants regardless of study condition
			3/7	• No differences between the intervention and control groups on alcohol consumption
			4/7	• Significant intervention effect; no specific intervention was decisively effective
			2/7	• Most positive findings only included patients aged 18 and older

*SBIRT* Screening, Brief Intervention and Referral to Treatment, *BI* Brief intervention, *ED* Emergency Department, *MI* Motivational Interviewing

Interventions also had mixed effectiveness on other alcohol-related outcomes (Table 3). For injury or re-injury, four reviews identified studies which reported lower rates of re-injury or significantly reduced alcohol-related injuries in the brief intervention group [19, 22, 24, 26]. Five reviews identified studies reporting reductions in driving offences or drink driving in the brief intervention group compared to control groups [18, 19, 22, 24, 26], though some of the reductions were not statistically significant. Two reviews found reductions in healthcare use, ED admission or re-admission or hospitalisations with a statistically significant difference between brief intervention and control groups in one study on the number of visits to ED [24] and positive effects of brief intervention on time to alcohol-related hospital events [26]. In those who were referred to treatment, there were mixed levels of participation and adherence to attending treatment sessions [18, 19, 26, 28]. Following brief intervention, one study found significantly higher numbers of patients attending referral treatments at four month follow-up [19], another study found positive effects of treatment adherence in youth who received a targeted BI [26]. One review identified mixed findings in relation to receiving further treatment following onsite brief advice, referral to post-discharge interventions, onsite extended brief intervention or a post-discharge letter without onsite intervention [28].

**Table 3:** Key findings for the effectiveness of interventions for outcomes related alcohol consumption

Author (year)	Intervention	Outcome	Number of studies	Results
Barata et al., (2017) [18]	SBIRT (no specified duration or sessions)	Alcohol-related negative consequences for physical and social consequences of alcohol use disorders	1/16	• Reduction in the concomitant use of marijuana and alcohol
			3/16	• Fewer injuries
			1/17	• Statistically significant changes in ‘trying to be careful while drinking’ (patients 18–21 years)
			1/17	• Beneficial effect in reducing drinking and driving (adolescents)
Diestelkamp et al., (2016) [19]	BI (max 60 min); max 3 sessions; 1 in ED	Alcohol-related risk behaviours; alcohol-related negative consequences; and/or seeking of further alcohol treatment or counselling		*Alcohol-related harm:*
			1/4	• Significant effect favouring the intervention group for reducing drinking and driving
			1/3	• Significant effect of the intervention on the quantity of alcohol-related injuries
			1/3	• Significant decline in moving violations in the intervention group at 6-months
				*Referral to treatment:*
			7/15	• Assessed whether participants accessed treatment or counselling following BI; referral rates in BI groups ranged 17% to 88% (mean 35.4%)
			1/4	• Significant intervention effects with patients in the intervention group reporting higher numbers in referral to treatment at 4-month follow-up
Kodadek et al., (2020) [22]	SBIRT (no specified duration or sessions)	Prevent or decrease reinjury; hospital readmission; alcohol-related offenses		*Rates of re-injury or ED/hospital readmission for re-injury:*
			3/3	• Lower rates of re-injury for patients receiving BI
			1/3	• Statistically significant lower rates of re-injury for patients receiving BI
				*Rates of alcohol related offences:*
			1/3	• Significant reduction in arrest for driving under the influence of alcohol after BI (1 study)
			2/3	• Fewer alcohol-related offenses after intervention but results did not meet statistical significance or had no control group for comparison (2 studies)
Landy et al., (2016) [24]	BI (5–60 min); single session; no additional booster sessions	ED visits/hospitalisations; alcohol-related injuries; alcohol-related risky behaviour		*ED admissions and hospitalisations:*
			1/5	• Statistically significant difference between BI and control groups on number of visits to ED
				*Alcohol-related injuries:*
			1/2	• Participants in BI condition were significantly less likely to experience an alcohol-related injury in the 6- or 12-months post-BI compared to the control group
			1/1	• No significant reductions in repetition of self-harm
				*Alcohol-related risky behaviour:*
			2/2	• BI found to be effective in reducing arrests and motor vehicle violations compared to the control group
Simioni et al., (2015) [28]	Intervention with referral to treatment	Treatment utilisation		*Onsite brief advice:*
			1/7	• Significant increase in receipt of specialist evaluation for further treatment at 6 months by injured patients compared to inactive control (1 study)
			2/7	• No relation between onsite brief advice and either participation in or completion of treatment at 3 and 12 months compared with active control conditions
				*Referral to post-discharge interventions:*
			2/7	• No increase in treatment utilization at 6 and 12 months after receipt of referral to post-discharge BIs compared with active control conditions
			1/7	• Significant increase in linkage to an assessment for treatment for patients who received a referral to a post-discharge 5-session case management intervention compared with a referral to a post-discharge 2-session BI and an active control condition
				*Onsite extended BI:*
			2/7	• Significant increase in treatment initiation and treatment adherence during the 12 months following an onsite extended compared with an inactive control condition
				*Post-discharge letter without onsite intervention:*
			1/7	• Significant increase in treatment initiation 6 months after a post-discharge letter without onsite intervention compared with an inactive control condition
Newton et al., (2013) [26]	BI (time limited); 1 or 2 sessions	Consequences of alcohol use; impact on healthcare use		*Impact of BIs on consequences related to alcohol/drug use:*
			2/2	• Targeted MI significantly reduced alcohol-related injuries up to 6 months after the ED visit, compared with brief advice or handout
			1/1	• Greater reduction in drinking and driving up to 6 months post-ED discharge
				*Impact of BIs on Healthcare Use:*
			1/1	• Increased likelihood of post-ED treatment adherence in youth who received a targeted BI that included referral and appointments with a community-based treatment agency compared to standard ED care; longer time to alcohol and other drug-related hospital event after discharge (not statistically significant)
Taggart et al., (2013) [29]	Standardised treatment to reduce alcohol intake	Alcohol-related harm	6/7	• All studies found reductions in alcohol-related harm in the intervention group; some between-group differences were not statistically significant
Yuma-Guerrero et al., (2012) [30]	BI (no specified duration or sessions)	Alcohol-related consequences	6/7	• Positive effects on consequences for all participants regardless of study condition (e.g., alcohol-related injuries; alcohol-related problems with friends, parents, police; drink driving; driving with an impaired driver)

*SBIRT* Screening, Brief Intervention and Referral to Treatment, *BI *Brief intervention, *ED *Emergency Department, *MI *Motivational Interviewing

#### Interventions for smoking

One study reported a mixture of self-report and/or the use of biomarkers to screen for smoking [34]; the other two studies did not provide information about the screening tools used (Additional File 5). Full details of participant characteristics, intervention content and comparator conditions are presented in Additional File 7. None of the studies provided data on participant characteristics. Intervention design and content for the intervention and control/comparator groups varied across studies included in the reviews. Interventions included brief advice, counselling or motivational interviewing, self-help materials and brochures, booster phone calls and referrals to telephone quit lines or other programmes [33–35]. Studies in two reviews included nicotine replacement therapies [33, 34]. Comparator groups included very brief advice, printed self-help materials and referrals to cessation phone line or local resources [33–35]. Table 4 shows the findings for the effectiveness of smoking interventions. Some studies included in the reviews found that smokers in the intervention group had higher abstinence rates than those in the comparator group [33–35], though one review reported findings were mostly non-significant between groups [34]. Meta-analyses in one study showed significant positive effects at 1- and 3-month follow-up and across pooled follow-up assessments, but a non-significant effect at 12 months, and when only the new studies were considered in the updated review the findings were non-significant [33]. In the earlier review, effects were positive and significant at 1 month but were non-significant at 3 and 6 months and when all follow-up assessments were pooled [35].

**Table 4:** Key findings for the effectiveness of interventions for smoking outcomes

Author (year)	Intervention	Outcome	Number of studies	Results
Lemhoefer et al., (2017) [33]	Smoking cessation interventions including MI, cessation advice, booster calls, NRT, materials and referral to telephone quit lines	Self-reported 7 days of tobacco-use abstinence	11 (pooled results: 4 new + 7 from Rabe et al., 2013)	• 1 month and 3 months after ETC (emergency-department initiated tobacco control), pooled 7-day point-prevalence abstinence was significantly higher in the ETC group compared to the control group; at 12-month follow-up, pooled results were not significant • For on-site motivational interviewing combined with booster telephone calls, pooled results showed higher point prevalence abstinence in ETC than in the control condition • Pooling only the 4 newly retrieved studies found the effect size was larger than in the previous meta-analysis
Pelletier et al., (2014) [34]	Smoking cessation interventions in an adult or paediatric ED setting including materials, brief advice, counselling, NRT, MI	Smoking cessation rate	12/13	• Differences in smoking cessation rates among intervention and comparison groups
			9/13	• Differences in cessation rates between compared groups were non-significant
			1/13	• No difference in self-reported quit rates between brief advice and motivational interviewing groups for paediatric patients; non-significant differences between the groups in self-reported smoking reduction rates
Rabe et al., (2013) [35]	Smoking cessation interventions including MI, referral to cessation programme, booster phone calls	Self-reported 7 days of tobacco-use abstinence	5/7	• Smoking abstinence higher in the intervention group compared with control group at follow-up
			3/7	• Positive significant effect of ETC on 7-day point prevalence tobacco abstinence at one month
			6/7	• Positive effects apparent at 3- and 6-month follow-up
			1/7	• Twelve-month data showed benefit of ETC compared with control condition
			7/7	• Benefit of ETC versus control condition on combined 7-day point prevalence tobacco abstinence
			5/7	• Sensitivity analyses: there was benefit of ETC on combined 7-day point prevalence tobacco abstinence when MI in the ED was followed by booster telephone calls

*ETC* Emergency Department-initiated tobacco control, *NRT* Nicotine Replacement Therapy

A summary of the evidence for the effectiveness of alcohol and smoking interventions in UEC is presented in Table 5.

**Table 5:** Summary of evidence for effectiveness of brief interventions delivered in UEC on alcohol and smoking behaviour

					**Quality Assessment (AMSTAR 2)**									
**Author (year)**	**Type of review**	**Study design**	**Intervention**	**Target age group**	**2. Methods**	**4. Search Strategy**	**7. Excluded studies listed**	**9. RoB – RCTs included**	**9. RoB – NSR included**	**11. MA—RCTs analysis**	**11. MA – NRS analysis**	**13. RoB—results**	**15. Publication bias / impact**	**Key conclusions from reviews**
**Alcohol interventions**														
Barata et al., (2017) [18]	SR	RCT	SBIRT^a^	All	N	N	N	N	NA	NA	NA	N	NA	Small reduction in alcohol use in low or moderate drinkers; short-term effect in reducing at-risk drinking
McGinnes et al., (2016) [25]	SR	MIX	Ultra BI; (10 min or less)	All	N	N	N	Y	NA	NA	NA	Y	NA	Limited effectiveness in reducing alcohol use in the short term
Schmidt et al., (2016) [27]	SR & MA	RCT	BI (5–40 min); 1 to 4 sessions	All	N	N	N	Y	NA	N	NA	N	Y	Evidence for very small effects on reducing alcohol consumption; no benefit from more intensive interventions
Elzerbi et al., (2015) [20]	SR & MA	RCT	BI (max 30 min); max 4 sessions	Adults (> 18)	N	N	Y	Y	NA	N	NA	Y	N	BI are associated with reducing alcohol consumption in hazardous and harmful drinkers at 6 and 12 months
Elzerbi et al., (2017) [21]	SR & MA	RCT	BI (max 45 min); max 4 sessions	Adults (> 16)	N	N	Y	Y	NA	N	NA	N	N	Non-injury specific studies are associated with a better response to BI than targeted injury studies
Kodadek et al., (2020) [22]	SR	MIX	SBIRT^a^	Adults	N	N	N	N	N	NA	NA	Y	NA	Conditional recommendation for SBIRT in emergency department/ trauma centre to reduce alcohol-related injury
Landy et al., (2016) [24]	SR	RCT	BI (5–60 min); single session	Adults (> 18)	N	N	N	N	N	NA	NA	Y	NA	BIs may not be effective in reducing alcohol consumption
Diestelkamp et al., (2016) [19]	SR	MIX	BI (max 60 min); max 3 sessions;	CYP (11–25)	N	N	N	Y	N	NA	NA	Y	NA	Heterogeneity in study design and effects limits conclusions on effectiveness of BIs in young patients
Kohler & Hofmann (2015) [23]	SR & MA	RCT	Motivational interviewing^a^	CYP (11–25)	N	N	N	N	NA	N	NA	Y	Y	MI as effective/more effective than other BI in emergency care to reduce alcohol consumption in young people
Newton et al., (2013) [26]	SR	RCT	BI (time limited); 1 or 2 sessions	CYP (11–25)	N	N	N	Y	NA	NA	NA	N	NA	Benefits of assessing ED-based BI to reduce alcohol use remains inconclusive due to variation in outcomes assessed / poor study quality
Taggart et al., (2013) [29]	SR	MIX	Standardised treatment	CYP (11–25)	N	N	N	N	N	NA	NA	N	NA	Interventions show promise but more research is needed to determine short- and long-term efficacy in college students
Yuma-Guerrero et al., (2012) [30]	SR	RCT	BI^a^	CYP (11–25)	N	N	N	N	NA	NA	NA	N	NA	It is unclear whether SBIRT is effective in reducing risky alcohol use in adolescent patients
**Smoking interventions**														
Lemhoefer et al., (2017) [33]	SR & MA	RCT	Smoking cessation interventions	All	N	N	N	N	NA	N	NA	N	Y	ED-initiated tobacco control is effective in promoting tobacco use abstinence up to 12 months post intervention
Pelletier et al., (2014) [34]	SR	MIX	Smoking cessation interventions	All	P-Y	N	N	Y	N	NA	NA	Y	NA	Most studies did not report significant differences in tobacco abstinence; those which did used MI-based interventions
Rabe et al., (2013) [35]	SR & MA	RCT	Smoking cessation interventions	All	N	N	N	N	NA	N	NA	N	Y	ED-initiated tobacco control with MI and booster phone calls showed a trend toward tobacco abstinence up to 12 months

^a^No details of the duration or number of sessions provided; *SR* Systematic review, *MA* meta-analysis, *RCT* randomised controlled trials only, *MIX* RCTs and other study types, *CYP* Children and young people, *NRS* non-randomised studies, *RoB* risk of bias, *N* No, *Y* Yes, *P-Y* partial yes, *NA* not applicable, *SBIRT* screening, brief intervention and referral to treatment, *BI* brief intervention, *MI* motivational interviewing, *ED* emergency department

#### Implementation

Findings are presented for two implementation-specific studies [31, 32] and from effectiveness studies which reported implementation-related outcomes (Table 6). Interventions were delivered by a wide range of staff with varying levels of experience. For example, researchers; clinical staff including physicians, nurses, psychologists, counsellors, or medical students; health promotion staff; or peer educators. Training for delivering interventions varied but where specified included reading materials and structured sessions [18], non-specified specialist training (3–30 h) [19] or extensive MI (motivational interview) training [23]. In alcohol studies, acceptance (of screening or intervention), participation, retention and adherence rates varied across studies [19, 25, 27, 32]. One study [25] noted that programmes which were delivered by ED clinicians had a higher rate of refusal and loss to follow-up compared with programmes where research assistants delivered the intervention. Only one smoking study reported an implementation outcome which was the time taken to deliver different types of intervention [34]. The shortest time was for a faxed referral (3 min), brief advice took on average 5 min and MI interventions took a mean time of 37 min. The main clinician reported barriers for delivering interventions were perceived lack of time, personal discomfort with concerns about the effect of the intervention on patient relationships, lack of knowledge about brief interventions and lack of resources e.g., screening tools and referral resources [31].

**Table 6:** Key findings for the implementation of interventions

Author (year)	Results
**ALCOHOL INTERVENTIONS: Intervention deliverers and training**	
Barata et al., (2017) [18]	• Physicians, medical students, mid-level providers, nurses, social workers, psychologists, community outreach workers and health promotion advocates • Staff training included reading materials about the assessment of adverse consequences of alcohol abuse, structured sessions to teach and practice the principles and techniques of SBIRT; ED staff nurses less fully engaged with SBIRT implementation when the ED was extremely busy
Diestelkamp et al., (2016) [19]	• Trained counsellors and psychologists or research staff most of whom had received special training (durations ranging from to 30 h)
Elzerbi et al., (2015) [20]	• Trained nurse, alcohol health worker, research assistants, research social worker, clinical ED staff, psychologist
Elzerbi et al., (2017) [21]	• Research social worker; Research assistants; Psychologist; Nurse clinician; Peer educators; Clinical ED staff; Alcohol health workers; Health promotion advocates
Gargaritano et al., (2020) [31]	• ED staff including physicians, nurses, directors, and coordinators
Kohler & Hofmann (2015) [23]	• Peer educators < 25 years old; bachelor's to master's level staff members with 1 to 2 years of experience; Research social workers; Bachelor’s and master’s level clinicians with previous experience; psychologist junior researchers (post-graduate or Master students) and one senior psychologist; Bachelor’s and master’s level interventionists with 1 to 2 years of clinical research experience • Training varied and included ‘extensive’ MI training; MI training (~ 24 h) that included readings, viewing videotapes, practicing MI techniques in training sessions led by doctoral and pre-doctoral supervisors, and participating in role-play interviews
Landy et al., (2016) [24]	• Physicians; nurses; social workers; emergency medical technician (EMTs); Residents; ED clinicians; peer educators; research social workers; research fellows; alcohol health workers; alcohol nurse specialist; psychologists; ED nurse; surgical nurses; surgeons; health promotion advocates; ED staff; degree level staff with 1–2 years’ experience; Master’s level clinicians and students; triage nurses; research staff
McGinnes et al., (2016) [25]	• Research social workers; Research assistant; Physicians; residents; physician associates; emergency physician; nurses; ED nurses and doctors; Staff nurses; research staff • Studies that used ED clinicians resulted in a high rate of refusal and significant loss to follow up; when research assistants performed the intervention, follow-up rates approached 80%
Newton et al., (2013) [26]	• Therapists, computers, peer educators, research team members
Schmidt et al., (2016) [27]	• External interventionists were employed (mainly research staff), Internal interventionists, ED personnel or trained nurses
Simioni et al., (2015) [28]	• Psychiatrist/social worker; Research assistants; ED Doctors; ED providers
**ALCOHOL INTERVENTIONS: Patient acceptance, participation, retention and adherence**	
Diestelkamp et al., (2016) [19]	*Participation rates (11 studies):* • On average, 68.8% of eligible youth agreed to take part in the BI • Participation rates ranged from 21.7% to 97.8% *Acceptance (3 studies):* • 75.9% of participants rated their overall impression of the intervention as ‘very good’, ‘good’ or ‘satisfactory’ immediately following the BI • Participants rated the BI as ‘helpful’; at 1-month follow-up, ratings were slightly lower for perceiving the BI as ‘helpful’ • 77.5% of participants reported they would recommend the BI to a friend in a similar situation; 60% of clinic staff rated the BI programme as being a valuable addition to ED standard care • Study participants rated counsellor’s perceived empathy, rapport and self-efficacy enhancement with generally positive ratings of 3.7–3.8 on a 4-point scale ranging from 1 (strongly disagree) to 4 (strongly agree)
Pedersen et al., (2011) [32]	*Acceptance:* • Screening acceptance rate: median 83% (range 31–98%) • Number of patients accepting intervention reported in all 28 studies; however, not all had information on number of eligible AUD patients • Acceptance rate for intervention among the eligible patients was 67% (21–96%) • Number needed to screen (NNS) to identify one eligible AUD patient = seven *Adherence:* • All but one trial conducted one or more follow-up visits; one-month follow-up visit—adherence rate was 62% (1 study); adherence rate after three months was 67% (54–96%) (10 studies); after six months 72% (45–89%) (15 studies) and 67% (27–92%) after twelve months
Schmidt et al., (2016) [27]	*Retention rates:* • Range 38 and 89.5%; median 75%
**ALCOHOL INTERVENTIONS: Barriers to delivering brief interventions**	
Gargaritano et al., (2020) [31]	• Lack of time (76% of studies), personal discomfort through healthcare worker concern about the effect on nurse–patient relationship, or patient demographics (60%), lack of knowledge (60%), lack of resources such as lack of screening tools and referral resources (52%), and patient presentation/condition such as time of injury, altered mental status, or unconscious state (44%)
**SMOKING INTERVENTIONS: Intervention time taken**	
Pelletier et al., (2014) [34]	*Intervention time:* • Time required for a faxed referral intervention alone (3 min) • Time required for brief advice, approximately 5-min brief advice intervention • Time required for motivational interviewing-based interventions, reporting a mean intervention time of 37 min • No study reviewed reported time required for pamphlet administration

## Discussion

To our knowledge this is the first umbrella review that has examined health promotion interventions for lifestyle behaviours in UEC settings. We aimed to provide an overview of the available review level evidence on the effectiveness and implementation for these interventions and present the implications for future research and clinical practice. Except for alcohol prevention, UEC settings have been under-researched and appear to be under-utilised for delivering health promotion activities. The only other lifestyle behaviour addressed was smoking for which there were few reviews. Effectiveness varied with some positive findings in favour of the brief intervention groups but comparisons between studies were difficult to due to heterogeneity in study design, target populations, intervention design, content and delivery mode in the studies included in the reviews, making it difficult to draw any clear conclusions. There was very little review level evidence on the implementation of SBIRT for lifestyle behaviours in UEC. Interventions have been delivered by a wide variety of staff who face barriers in undertaking this role [31]. Nonetheless, alcohol brief interventions appear to be acceptable to patients attending UEC [32].

All studies took place in emergency departments or trauma centres. None of the studies included in the reviews investigated the use of other UEC settings, such as urgent care centres, minor injury units and walk-in centres, which could provide further opportunity for health promotion interventions. Indeed, the provision of advice and information about healthy lifestyles was intended as a key feature of walk-in centres in England when they were first established [36], although this has not been implemented [37]. The evidence available for health promotion and the effectiveness of interventions delivered in UEC settings on changing patient behaviour varies according to lifestyle behaviour topic. There is substantial review level evidence for the effectiveness of brief interventions for alcohol prevention in UEC settings across different age groups, but limited review level evidence for smoking interventions. No review level evidence was identified for physical activity or diet and nutrition. Evidence suggests this may be because staff are worried about discussing these topics with patients due to seeming insensitive or stigmatising patients, so few studies or interventions have been conducted in these areas despite patients reporting they are most interested in these topics [3].

Reviews of alcohol interventions in UEC as well as individual studies of alcohol interventions have been heterogeneous in study design, target population, screening tools used, intervention implementation (including who delivers the intervention), intervention content and design, and the outcomes studied. As a result, it is challenging to draw any clear conclusions about the effectiveness of these interventions. Interventions which target all age groups may have some small or very small positive effects in the short-term from brief or ultra-brief interventions [18, 25, 27] which supports continued implementation of these interventions in practice. However, further research is required to identify the optimal intervention design and strategies to encourage a longer-term intervention effect. In contrast, brief interventions targeting children, young people and adolescents were mostly inconclusive [19, 26, 29, 30] with the exception of those that included motivational interviewing which showed positive effects [23]. Interventions targeting alcohol consumption in children and young people may therefore benefit from including a motivational interviewing component and this warrants further investigation. Review level evidence for interventions targeting adults suggests that a single session of brief advice may be insufficient to reduce alcohol consumption [24]. In contrast, reviews which included brief interventions of greater than one session were more likely to report positive effects of the interventions on alcohol consumption [20, 22], and this may be more effective in specific target groups [21]. The optimal number of sessions of a brief intervention along with the effectiveness of interventions when targeting specific population groups also requires further research. It is worth noting that comparator groups have often received some form of intervention (for example screening only, a brief conversation, or printed materials) which appears sufficient to impact on alcohol consumption in some studies. This requires further investigation as minimal interventions such as these could be important for designing future effective and cost-effective interventions in the UEC setting placing limited burden on staff. The findings for impact on alcohol-related outcomes were mixed with generally only small numbers of studies demonstrating a positive impact on reductions in alcohol-related injuries, hospitalisations and drink driving. Further research is needed to optimise interventions to address these related outcomes and reduce the burden of such outcomes on the health service and society.

For smoking, again, there was considerable heterogeneity in study design, and intervention design and content across studies included in the reviews. However, the evidence suggests interventions delivered in UEC to reduce smoking can be effective. One of the included reviews [33] was an update of a previous review [35] and both reported positive effects in reducing tobacco use following brief interventions, which included motivational interviewing and booster telephone calls. A further review reported that studies were only effective when motivational interviewing was included [34]. As for alcohol interventions, motivational interviewing and brief interventions which include more than one session may be important characteristics of effective interventions for reducing smoking delivered in UEC settings.

Except for alcohol, UEC settings have been under-researched and remain underutilised for health promotion interventions addressing the broad spectrum of lifestyle behaviours. Whilst clinical guidelines have been published in the UK for interventions targeting alcohol including EDs [5], and for smoking cessation more broadly [7], the guidance is embedded within general guideline documents and thus UEC healthcare professionals may not be aware of their existence or relevance to their practice. Developing UEC setting-specific health promotion guidelines and policy across the spectrum of lifestyle behaviours may be needed to raise its profile, to make it accessible for healthcare organisations, commissioners, managers, and healthcare professionals and to ensure UEC healthcare professionals are aware that it is part of their role. This might help to encourage effective implementation via changes to ED curricula, training and service delivery by integrating SBIRT into ED workflows. UEC healthcare professionals could then increase their contribution to health promotion as a natural part of their role.

There was very limited review level evidence on the implementation of health promotion interventions in UEC settings, with only two reviews published which were specifically related to alcohol interventions. These reviews showed that alcohol screening and interventions are acceptable to patients in emergency care, and that most will complete the programme [32]. However, there are barriers for clinicians in delivering such interventions which relate to lack of knowledge, lack of time and resources and personal discomfort with regard to impact on the relationship with patients [31]. These barriers have also been reported in a review of wider health promotion activities in UEC settings [3] and will need to be addressed to increase the frequency and impact of health promotion interventions delivered in UEC. There was generally minimal reporting of aspects of implementation in effectiveness reviews. This was limited to who delivered the intervention (a wide range of staff including researchers and clinical staff with varying levels of training) and brief descriptions of intervention content (which varied in the length and number of brief conversations, on whether the provision of educational materials were via print or text message, and on whether follow-up or booster sessions were delivered face-to-face or by telephone). More detailed process evaluations are required to identify optimal implementation characteristics for health promotion interventions in UEC settings. This might include who delivered the intervention, staff training provided, delivery mode, timing, frequency and duration, content, barriers and facilitators, staff and patient views on acceptability, feasibility, and integration of interventions into routine clinical practice.

## Strengths and limitations

A strength of our study is that we attempted to consider a broad definition of health promotion in UEC settings across multiple lifestyle behaviours rather than just single behaviours. Although this was a rapid review, we followed rigorous, systematic approaches using the PRISMA 2020 guidelines [16] and completed a quality assessment using AMSTAR 2 [17]. Due to time constraints, we only included reviews sourced from two databases, however, the databases used were likely to contain most studies relating to UEC research or health promotion in clinical settings. Only systematic reviews or meta-analyses were included, and therefore it is possible that some pertinent evidence may have been excluded. We assessed the critical domains of AMSTAR 2 rather than using the full instrument due to time limitations which enabled us to gain an overview of the quality of the included reviews. The quality of included reviews varied but was generally poor which may limit the findings from this study. The review was undertaken by one researcher which may have introduced bias in selection and retrieval of papers, data extraction and quality appraisal. However, risk of bias was mitigated by involvement of additional researchers to verify 10% of included studies and cross-check 20% of data extraction. In addition, the study team included clinical colleagues from an ED who helped to identify the need for this study and to interpret the findings. We specifically searched for UEC related terms, and we may therefore have excluded papers on brief interventions in UEC settings if they were included under the umbrella of primary health care or general medical settings/hospitals. Due to time constraints and the nature of this rapid review we included review papers from January 2010 onwards, however many of the reviews included individual studies from pre-2010 so we are likely to have captured much of the evidence in our study. As the study team are only English speaking, we were unable to include studies written in other languages, we may therefore have excluded some relevant review papers from our study. Heterogeneity in the purpose of the reviews, varying inclusion and exclusion criteria of reviews and heterogeneity in the studies included in reviews meant it was not possible to combine data and conduct a meta-analysis and made it challenging to draw any clear conclusions with regards to effectiveness. Searching for individual studies may have yielded greater insight into the state of the evidence for health promotion in UEC settings for a broader range of behaviours. We did not include drug/substance misuse, mental health or sexual behaviour in our review, these areas may also benefit from SBIRT delivered in UEC settings.

### Implications for future research

There is a need for further research assessing the effectiveness and implementation of health promotion interventions in UEC settings including EDs and trauma centres as well as urgent care centres, minor injury units and walk-in centres. In particular, an assessment of the available evidence for the effectiveness of interventions for physical activity and diet and nutrition in UEC is required, as no reviews were identified in the current study. Similarly, research is needed on effective approaches to implementation of interventions across all lifestyle behaviours in this challenging healthcare environment. Standardisation in study designs, measurement tools, study outcomes, target populations, screening tools, intervention content/design, intervention implementation, the content of control/comparison group interventions (which often included a brief verbal intervention or written materials) and follow-up time periods would facilitate future comparisons between studies and synthesis of the evidence. Standard care was sometimes used as a control/comparator, but the nature of the standard care is often not described making it difficult to assess the dose of intervention which patients in the control group received. Clear descriptions of the interventions delivered in the intervention and control/comparator groups, which may benefit from the use of the Template for Intervention Description and Replication (TIDieR) checklist [38], as well as replication studies, would also facilitate comparisons between interventions and study findings. This would help to build a more robust and useful evidence base for the effectiveness of health promotion interventions in UEC settings and increase opportunities for meta-analysis and would support the inclusion of teaching on effective approaches in the medial curricula.

Further research is needed to determine the optimal design, duration, content, and delivery mode for health promotion interventions in UEC settings. This includes answering questions such as 1) which patients should be included (a universal approach or specific target groups); 2) whether screening or the provision of written information is enough to initiative behaviour change and in which patients; 3) who is best placed to deliver interventions and when is the best time to have a conversation; 4) whether face-to-face interventions are most effective or whether digitalised interventions could be used instead to reduce burden on staff and resources; 5) what the optimal duration and content of a brief intervention is and whether very brief interventions (less than 5 min) would be sufficient to change behaviour; 6) whether follow-up or additional support is needed to maximise and sustain the effectiveness of the intervention in the long-term; 7) which patients require additional support and what format this should take (booster sessions or reminders); and 8) whether the SBIRT process could be conducted in the waiting room prior to consultation, where patients complete digital screening tools and educational materials are presented or sent to an e-mail address, or patients are directed to online resources or support services.

Process evaluations of interventions are needed to help understand the implementation of interventions (including for example implementation determinants and outcomes such as reach, dose, acceptance, feasibility, and costs) and explain study findings. Further research is needed to investigate the implementation and impact of referring patients to treatment outside of the UEC setting, the uptake of treatment and what type of treatment is most effective in initiating and maintaining behaviour change in this population. Finally, more research is needed to understand the uptake of health promotion into UEC settings, whether a comprehensive but consistent approach addressing multiple lifestyle behaviours could be effective, how interventions can be implemented in practice, what is needed at the individual, environmental, organisational and policy levels to support implementation and maximise the cost-effectiveness of such interventions.

The quality of the reviews included in this study was assessed and was generally found to be poor, partly due to lack of reporting of methodological details in the review publications. Future systematic reviews and meta-analyses should consider the quality of their review methodology and ensure sufficient detail is provided in publications for an accurate quality assessment to be made. In addition, for future systematic reviews and meta-analyses, it may be necessary to consider more stringent inclusion criteria. This might include targeting studies which have used interventions with more specific designs and components or grouping studies with more similar intervention designs and components together, to facilitate synthesis and comparisons of effectiveness between studies. Researchers should ensure that a quality assessment of studies is included in future reviews.

### Implications for clinical practice and policy

Based on the review level evidence in this study, UEC settings appear to have been underutilised for health promotion interventions. However, there is some evidence that alcohol and smoking interventions may be effective, at least in the short-term and albeit with small effects, for some population groups, particularly when motivational interviewing is included along with additional sessions following the initial brief advice given in the UEC setting. Whilst there is some evidence that EDs in the UK screen patients for alcohol consumption and may provide some form of intervention or referral to treatment [39], there is potential to increase the use of SBIRT to address multiple lifestyle behaviours including alcohol, smoking, physical activity, and diet and nutrition. There may also be potential to address other areas such as drug misuse, mental health, and sexual health, however, these were outside the scope of this review. Actions need to be taken to raise the profile of UEC settings role in the health promotion and prevention agenda at multiple levels. This might include organisational change and prioritisation by senior leadership to implement such interventions, raising staff awareness of their role in health promotion, supporting staff to undertake this role, environmental change to prompt and remind staff about health promotion screening, intervention and referral (e.g., changes to IT systems, inclusion in clinical pathways), educating staff to improve skills and knowledge for delivering interventions and making referrals, and collecting and monitoring data on the number of patients screened, the number and type of interventions delivered and referrals made. Nationally, setting-specific policy and guidance for health promotion in UEC should be developed to raise awareness of the potential of this setting for improving population health, and to raise the profile of these types of interventions for key stakeholders, commissioners and decision makers who are planning and managing healthcare delivery in UEC.

## Conclusions

UEC settings offer a unique opportunity to deliver health promotion interventions with potential to reach a large proportion of the population who are at risk of ill health from unhealthy lifestyle behaviours and who may not access primary care. Findings from this review suggest UEC settings have been under-researched and under-utilised for delivering health promotion activities, except for alcohol prevention, which has been well studied, and a small number of studies addressing smoking. There is considerable heterogeneity across studies in design, populations, intervention design and content and the outcomes assessed. However, the evidence suggests alcohol and smoking interventions delivered in UEC settings may be effective at least in the short-term and in specific population groups, particularly when motivational interviewing is included along with booster sessions. Further research is needed to determine the optimal intervention design and content for promoting healthy lifestyle behaviours which is suitable for implementation in UEC settings. In addition, changes are needed in clinical practice to deliver health promotion activities requiring increased staff training, thoughtful integration into service delivery and supportive policies. Ultimately, this could reinforce health promotion in UEC settings and could make a difference to population health and wellbeing by reducing ill health, improving individual quality of life, reducing health inequalities and reducing pressure on healthcare services and resources.

## Supplementary Information

**Additional file 1: Table A1.** Key terms and combinations of search terms used in literature search.

**Additional file 2: Table A2.** Reasons and references for studies excluded at abstract/full text review stage.

**Additional file 3: Table A3.** Inclusion criteria for intervention studies (PICO).

**Additional file 4: Table A4a and A4b.** AMSTAR 2 quality assessment.

**Additional file 5: Table A5.** Screening tools used in intervention studies.

**Additional file 6: Table A6.** Participant characteristics, intervention design and content, and comparator/control conditions: alcohol interventions.

**Additional file 7: Table A7.** Participant characteristics, intervention design and content, and comparator/control conditions: smoking interventions.

## Data Availability

All data extracted from reviews during this study are included in this published article and additional files.

## Abbreviations

AHW: Alcohol Health Worker
AUD: Alcohol Use Disorder
BI: Brief intervention
COM-B: Capability, Opportunity, Motivation-Behaviour
ED: Emergency department(s)
ETC: Emergency department-initiated tobacco control
GPs: General practitioners
IT: Information Technology
PICO: Population, Intervention, Comparator/control conditions, Outcomes
RCT: Randomised controlled trial
RR: Relative risk
SBIRT: Screening, Brief Intervention and Referral to Treatment
SMD: Standardised mean difference
UEC: Urgent and emergency care
USA: United States of America
