# Supplementary material for: Effectiveness and implementation of interventions for health promotion in urgent and emergency care settings: an umbrella review
Source: BMC Emerg Med. 2023 Apr 6;23:41. doi: 10.1186/s12873-023-00798-7 (PMC10080902; doi:10.1186/s12873-023-00798-7)
Supplement: Supplementary file 1 — Additional file 1: Table A1. Key terms and combinations of search terms used in literature search. [file 12873_2023_798_MOESM1_ESM.docx]

**Additional File 1**

**Table A1. Key terms and combinations of search terms used in literature search**

| **Set** | **Search terms** | **Fields searched** |  |
| --- | --- | --- | --- |
|  |  | **PubMed** | **Embase (OVID)** |
| #1 | “emergency care” OR “urgent care” OR “emergency department*” OR “accident and emergency” OR “emergency medicine” OR “trauma cent*” OR “trauma unit*” OR “emergency room” OR “emergency nurse*” OR “emergency physician*” OR “emergency medical service*” OR “hospital emergency service*” | Title or abstract | Keyword |
| #2 | “brief intervention*” OR “brief advice” OR “simple advice” OR “intervention*” OR “minimal intervention*” OR “SBIRT” OR “educat*” OR “counsel*” OR “interview*” OR “program*” OR “screening” OR “referral*” OR “teachable moment*” OR “feedback” OR “conversation*” | Title or abstract | Keyword |
| #3 | “health promotion” OR “health improvement” OR “prevent*” OR “life style” OR “lifestyle” OR “risk factor*” OR “behaviour*” OR “health education” OR “health coach*” | Title or abstract | Keyword |
| #4 | “systematic review” OR “meta-analysis” | Title | Title |
| **Combined searches** | |  |  |
| S1 | #1 and #2 and #4 |  |  |
| S2 | #1 and #3 and #4 |  |  |
| S3 | #1 and #2 and #4 OR #1 and #3 and #4 |  |  |
