# Supplementary material for: Effectiveness and implementation of interventions for health promotion in urgent and emergency care settings: an umbrella review
Source: BMC Emerg Med. 2023 Apr 6;23:41. doi: 10.1186/s12873-023-00798-7 (PMC10080902; doi:10.1186/s12873-023-00798-7)
Supplement: Supplementary file 2 — Additional file 2: Table A2. Reasons and references for studies excluded at abstract/full text review stage. [file 12873_2023_798_MOESM2_ESM.docx]

**Additional File 2**

**Table A2. Reasons and references for studies excluded at abstract/full text review stage**

| **Reason** | **References** |
| --- | --- |
| Duplicate study listed with incorrect first author (2 studies) | - Rolland incorrectly listed as first author in Simioni, N., Rolland, B., & Cottencin, O. (2015) Interventions for Increasing Alcohol Treatment Utilization Among Patients with Alcohol Use Disorders from Emergency Departments: A Systematic Review, *Journal of Substance Abuse and Treatment*, 58, 6-15 - Mabood incorrectly listed as first author in Newton, A.S., Dong, K., Mabood, N., Ata, N., Ali, S., Gokiert, R., Vandmeer, B., Tjosvold, L., Hartling, L., & Wild, T.C. (2013) Brief emergency department interventions for youth who use alcohol and other drugs: a systematic review, *Pediatric Emergency Care*, 29(5), 673-684 |
| Only reviewed measurement tools (3 studies) | - Jones, L.A. (2011) Systematic review of alcohol screening tools for use in the emergency department. *Emergency Medicine Journal*, 28(3), 182-191 - Newton, A.A., Gokiert, R., Mabood, N., Ata, N., Dong, K., Ali, S., Vandmeer, B., Tjosvold, L., Hartling, L., & Wild, T.C. (2011) Instruments to detect alcohol and other drug misuse in the emergency department: a systematic review, *Pediatrics*, 128(1), e180-192 - Mabood incorrectly listed as first author in Newton et al., (2011) |
| Assessed the impact of an educational intervention on staff rather than an intervention for patients (2 studies) | - Clarke, D.E., Gonzalez, M., Pereira, A., Boyce-Gaudreau, K., Waldman, C., & Demczuk, L. (2015) The impact of knowledge on attitudes of emergency department staff towards patients with substance related presentations: a quantitative systematic review protocol. *JBI Database System Rev Implement Rep,* 13(10), 133-145 - Gonzalez, M., Clarke, D.E., Pereira, A., Legare, C., Boyce-Gaudreau, K., Demczuk, L., & Waldman, C. (2017) The impact of educational interventions on attitudes of emergency department staff towards patients with substance-related presentations: A quantitative systematic review. *JBI Database System Rev Implement Rep,* 15(8), 2153-2181 |
| Unable to separate out results of study specifically for Emergency Department settings (1 study) | - Bray, J.W., Cowell, A.J. & Hinde, J.M. (2011) A systematic review and meta-analysis of health care utilization outcomes in alcohol screening and brief intervention trials. *Medical Care,* 49(3), 287-294 |
| Insufficient data provided for included studies to enable data extraction and interpretation of findings for Emergency Department settings and target behaviours | - Tanner-Smith, E.E., Parr, N.J., Schweer-Collins, M., & Saltz, R. (2022) Effects of brief substance use interventions delivered in general medical settings: a systematic review and meta-analysis. *Addiction,* 117, 877-889 |
| Review did not target one-to-one or face-to-face interventions | - Schofield, B., Rolfe, U., McClean, S., Hoskins, R., Voss, S & Benger, J. (2022) What are the barrier and facilitators to effective health promotion in urgent and emergency care? A systematic review. *BMC Emergency Medicine*, 22:95 |
