# Supplementary material for: Effectiveness and implementation of interventions for health promotion in urgent and emergency care settings: an umbrella review
Source: BMC Emerg Med. 2023 Apr 6;23:41. doi: 10.1186/s12873-023-00798-7 (PMC10080902; doi:10.1186/s12873-023-00798-7)
Supplement: Supplementary file 3 — Additional file 3: Table A3. Inclusion criteria for intervention studies (PICO). [file 12873_2023_798_MOESM3_ESM.docx]

**Additional File 3**

**Table A3. Inclusion criteria for intervention studies (PICO)**

| **First author (year)** | **Study population** | **Intervention(s)** | **Comparison/control** | **Outcome(s)** |
| --- | --- | --- | --- | --- |
|  | | | | |
| **Effectiveness studies (Alcohol)** | | | | |
| Barata et al., (2017) | All ages with known or suspected alcohol use disorders (AUD) or alcohol-related injures | Screening, brief intervention, referral and treatment (SBIRT) | No data | *Primary*: Reduction of alcohol consumption  *Secondary*: Reducing alcohol-related negative consequences for physical and social consequences of AUD |
| Diestelkamp et al., (2016) | Aged between 12 and 25 years and treated in an emergency care setting (inpatient or outpatient) following an alcohol-related event | The intervention is a brief intervention (maximum 60 min) consisting of a maximum of 3 sessions with a minimum of 1 session delivered in the ED; The intervention is focused on alcohol use and is delivered in person. | The control condition consists of no treatment, standard care, an intervention other than a BI, or a BI of different intensity. | Outcome measures address one or more of the following: alcohol consumption, alcohol-related risk behaviours, alcohol-related negative consequences and/or seeking of further alcohol treatment or counselling.  For additional evidence: Participation rates, acceptance, implementation and intervention effects on alcohol use, alcohol-related harm and referral to treatment. |
| Elzerbi et al., (2015) | Aged 18-64 years old; participants not seeking treatment for alcohol and met a minimum criterion of hazardous or harmful drinking (hazardous and harmful drinking was understood as regular average consumption of 20–40 g and >40 g of alcohol per day for women and 40–60 g and >60 g per day for men. | Brief Intervention was understood as opportunistic screening and early intervention (no more than four sessions, each session lasting no longer than 30minutes) delivered by non-specialist personnel carried out in non-specialist settings | Comparator control groups varied from ‘screening only’, ‘assessment only’ to ‘treatment as usual’ ‘evaluation only’ or ‘minimal intervention’ (such as the provision of an information leaflet). | Primary outcome measure was quantity of alcohol consumed per week at 6- and 12-month follow-up. |
| Elzerbi et al., (2017) | Aged 18-64 years old; participants not seeking treatment for alcohol and met a minimum criterion of hazardous or harmful drinking (hazardous and harmful drinking was understood as regular average consumption of 20–40 g and >40 g of alcohol per day for women and 40–60 g and >60 g per day for men. | Brief intervention was understood as opportunistic screening and early intervention (brief intervention was defined as no more than 4 sessions, each session lasting no longer than 45 minutes and delivered face-to-face, by short message service, detailed health information workbooks, over the telephone or electronically) and was delivered by non-specialist personnel and carried out in non-specialist settings. | Comparator control groups varied from ‘screening only’, ‘assessment only’ to ‘treatment as usual’ ‘evaluation only’ or ‘minimal intervention’ (such as the provision of an information leaflet). | The primary outcome measure was grams of alcohol consumed at less than or equal to 5-, 6-, and 12-month follow-up, in which 14 g of alcohol was taken to be the equivalent of one “standard” drink. |
| Kodadek et al., (2020) | Adult trauma patients inclusive of race, ethnicity, sex, and other demographic characteristics. | Any ED, trauma centre, or hospital-based alcohol screening with brief intervention and/or referral to treatment (SBIRT) for appropriate patients | Usual trauma care | Re-injury, hospital readmission, alcohol-related offenses, and/or alcohol consumption |
| Kohler & Hofmann (2015) | Studies included at least some, young people of age 18 years or younger with existing alcohol use problems, who were admitted to an emergency care unit alcohol positive, with an alcohol-related trauma, or with a history of elevated alcohol consumption; Studies were excluded if they allowed participants older than 25 years. | Motivational Interviewing (MI) or motivational enhancement therapy (MI plus feedback) that targeted alcohol consumption | Another or no intervention | Changes in alcohol consumption (drinking frequency or drinking quantity) |
| Landy et al, (2016) | Adult sample with alcohol misuse (majority of participants between 18 and 65 years of age). | Brief Intervention - a single-session intervention, typically lasting between 5 and 30 minutes; a few of the BIs described in studies lasted as long as 60 minutes. Studies were excluded if they included booster sessions following the delivery of a BI and did not report the effects of the initial BI alone. | The BI condition was compared to a control condition. Sometimes participants in the control condition were screened for at risk drinking and then given an information booklet or printed resources. Other times, they received an assessment or usual care. Some studies compared the BI to extended counselling, and others did not have a comparison condition at all | Four main outcomes of interest: reduction in alcohol consumption, ED visits/ hospitalizations alcohol-related injuries, and alcohol-related risky behaviour. |
| McGinnes et al., (2016) | Adults and adolescents with at-risk drinking behaviour, dysfunctional drinking patterns or symptoms of an alcohol-related disorder attending an ED. | Ultra-Brief Intervention (defined as any face-to-face interaction of 10 min or less or any non face-to-face intervention involving technology); Intervention included screening for alcohol use, intervention by means of feedback, negotiation and goal setting, provision of information (pamphlet, computer or phone) and referral for treatment where clinically appropriate. Interventions were carried out by a physician or a member of the physician’s multidisciplinary team, a member of the allied health team or a research team. | Control groups received screening only, assessment only or minimal intervention that included the provision of written information or standard care. | Change in the frequency of alcohol use, quantity of alcohol consumed, including binge drinking, or change in the frequency of ED representation over any time period. |
| Newton et al., (2013) | Patients 19 years or younger (post hoc decision to include studies that predominantly included the specified age range (indicated by reported mean age) but extended into early adulthood (up to 21 years) with harmful or hazardous alcohol consumption. | Effect of BIs aimed at improving outcomes related to harmful and hazardous alcohol and other drug use and associated morbidities in patients 19 years or younger. Brief interventions (BIs) were defined as time-limited efforts (1 or 2 contacts) that follow screening for hazardous and harmful behaviours. | No restrictions were placed on comparison interventions (control groups) | Main treatment outcome related to harmful and hazardous alcohol use (e.g., injuries or high-risk behaviour, frequency of use). |
| Schmidt et al., (2016) | All age groups (including intoxicated and/ or injured patients) were included, except for studies which focused solely upon adolescents (i.e. excluded patients > 17 years). | Consisting of one to four BI sessions, each lasting 5–30 minutes (or 40 minutes, if only one session was provided). | Not specified | Changes from baseline in consumption quantity, intensity and number of heavy drinking episodes were assessed at 3-, 6- and 12-month follow-up |
| Simioni et al., (2015) | Adults 18 or older with alcohol use disorders (but not alcohol dependent) admitted to ED for any reason | Patients in intervention group received a referral | Any control/comparison group was eligible | Reduce alcohol use and related consequences |
| Taggart et al., (2013) | College drinkers: 18-20 year old alcohol users in ED or acute care setting where intervention regarding alcohol use was attempted. | Intervention was defined as a standardized treatment designed to reduce alcohol intake. | Not specified | Changes in alcohol intake patterns  Reductions in alcohol-related harm |
| Yuma-Guerrero et al., (2012) | Included participants within the adolescent age range (i.e., 11–21 years of age). | Brief interventions for alcohol use, conducted in the U.S. | Not specified | Not specified but included patients receiving treatment for an alcohol-related event, an injury or illness. |
| **Effectiveness studies (Smoking)** | | | | |
| Lemhoefer et al., (2017) | Emergency Department patients of any age who were current smokers and who were offered a tobacco control intervention. | Motivational interviewing or counselling on site in combination with referral to outpatient treatments or to telephone quit lines | The control group could be usual care (receipt of brochures, self-help material, information leaflets on state smokers’ quit lines, or any less intensive program such as brief advice only or no material or advice at all) | Self-reported 7 days of tobacco-use abstinence (point-prevalence tobacco-use abstinence, measured at least once during follow-up) |
| Pelletier et al., (2014) | Adult or paediatric ED setting | Smoking cessation interventions performed or initiated in an adult or paediatric ED setting | Not specified | *Primary*: smoking cessation rate *Secondary*: all-cause mortality, patient satisfaction, clinician time spent, non-clinician time spent, and cost per quit |
| Rabe et al., (2013) | ED patients of any age who were current smokers | Smoking cessation interventions in ED patients who smoke (preventive services such as screening and brief interventions delivered in the ED to promote smoking cessation such as motivational interviewing and/or referral to outpatient treatments) | The treatment in the control group could be usual care or any less intensive program such as brief advice only | Number of abstinent smokers at follow-up (point-prevalence tobacco-use abstinence, measured at all available follow-up times) |
|  | | | | |
| **Implementation studies (Alcohol)** | | | | |
| Gargaritano et al., (2020) | Clinicians working in hospital settings including inpatient wards and the emergency department | Defined as: short and structured discussion-based interventions aimed to support patients in reducing their consumption of alcohol | Not applicable | Clinician reported barriers to the provision of alcohol screening and brief interventions in hospital settings were included; categorised using the COM-B model (Capability, Opportunity, Motivation – Behaviour) |
| Pedersen et al., (2011) | Adult elective or acute patients with AUDs treated in a surgical or emergency department | All alcohol intervention programs focusing on alcohol reduction or cessation. Interventions could be brief or intensive, including programs with pharmaceutical interventions for alcohol withdrawal and relapse prophylaxis. | Control groups were defined as assessment of AUDs only or treatment as usual. | Screening acceptance rate = Number screened for AUDs/Total patient population  Intervention acceptance rate = Number participating in intervention/Number of eligible AUD patients fulfilling study inclusion criteria  Adherence rates = Number of patients at follow-up(s)/Number of patients accepting intervention  Numbers needed to screen (NNS) = 1/(number of eligible/total patient population) and 1/(number accepting intervention/total patient population) |
