# Supplementary material for: Effectiveness and implementation of interventions for health promotion in urgent and emergency care settings: an umbrella review
Source: BMC Emerg Med. 2023 Apr 6;23:41. doi: 10.1186/s12873-023-00798-7 (PMC10080902; doi:10.1186/s12873-023-00798-7)
Supplement: Supplementary file 4 — Additional file 4: Table A4a and A4b. AMSTAR 2 quality assessment. [file 12873_2023_798_MOESM4_ESM.docx]

**Additional File 4**

**Table A4a. Summary of findings from AMSTAR 2 (Critical Domains) assessment of included reviews**

| **Q** | **AMSTAR 2 Question** | **Summary of findings** |
| --- | --- | --- |
| 2 | Did the report of the review contain an explicit statement that the review methods were established prior to conduct of the review and did the report justify any significant deviations from the protocol? | The included reviews generally did not contain explicit statements that the review methods were established prior to the conduct of the review, with the exception of one study. Only five out of the eighteen includes studies registered or used a previously published study protocol. Only two studies reported protocol deviations. |
| 4 | Did the review authors use a comprehensive literature search strategy? | All studies searched at least two databases and provided the key word and/or search strategy. However, only one study justified any publication restrictions. Fifteen of the eighteen studies searched the reference lists of included studies but only four searched trial registries, and three consulted experts in the field. Four studies searched for grey literature. All studies conducted the searches within 24 months of completion of the review. |
| 7 | Did the review authors provide a list of excluded studies and justify the exclusions? | Only three studies provided a list of excluded studies with reasons. Many studies provided a summary of the reasons for exclusion, but these were not linked to individual studies. |
| 9 | Did the review authors use a satisfactory technique for assessing the risk of bias (RoB) in individual studies that were included in the review? | Six studies used the Cochrane Collaboration Risk of Bias Tool; two studies used the risk of bias table in Review Manager; Other studies used the Critical Appraisal Skills Programme RCT checklist, the Quality Assessment Tool for Quantitative Studies, the Crowe Critical Appraisal Tool, GRADE or 8 questions from an unspecified tool. Four studies did not use a Risk of Bias Tool.  Risk of bias was satisfactorily assessed in eight out of the eighteen studies. |
| 11 | If meta-analysis was performed, did the review authors assess the potential impact of RoB in individual studies on the results of the meta-analysis or other evidence synthesis? | Six reviews included a meta-analysis. Of these, none of the studies specifically justified combing the data in a meta-analysis. All used a weighted technique to combine study results. Three investigated causes of heterogeneity. |
| 13 | Did the review authors account for RoB in individual studies when interpreting/ discussing the results of the review? | Four reviews included only low risk of bias studies, a further three provided some discussion of the likely impact of RoB in the results. |
| 15 | If they performed quantitative synthesis did the review authors carry out an adequate investigation of publication bias (small study bias) and discuss its likely impact on the results of the review | Four of the six reviews which included a meta-analysis assessed publication bias. |

**Table A4b. Findings from AMSTAR 2 (Critical Domains) assessment of included reviews**

|  |  | Alcohol | | | | | | | | | | | | | Smoking | | | Implementation | |
| --- | --- | --- | --- | --- | --- | --- | --- | --- | --- | --- | --- | --- | --- | --- | --- | --- | --- | --- | --- |
| **AMSTAR 2 Question** |  | Barata et al., (2017) | Diestelkamp et al., (2016) | Elzerbi et al., (2015) | Elzerbi et al., (2017) | Kodadek et al., (2020) | Kohler & Hofmann (2015) | Landy et al, (2016) | McGinnes et al., (2016) | Newton et al., (2013) | Schmidt et al., (2016) | Simioni et al., (2015) | Taggart et al., (2013) | Yuma-Guerrero et al., (2012) | Lemhoefer et al., (2017) | Pelletier et al., (2014) | Rabe et al., (2013) | Gargaritano et al., (2020) | Pedersen et al., (2011) |
|  | Systematic Review (SR) or Meta-Analysis (MA) | SR | SR | SR/ MA | SR/ MA | SR | SR/ MA | SR | SR | SR | SR/ MA | SR | SR | SR | SR/ MA | SR | SR/ MA | SR | SR |
|  | RCTs only (RCT) or Mixed study designs (MIX) | RCT | MIX | RCT | RCT | MIX | RCT | RCT | MIX | RCT | RCT | MIX | MIX | RCT | RCT | MIX | RCT | MIX | MIX |
| 2. Did the report of the review contain an **explicit statement that the review methods were established prior to conduct of the review** and did the report justify any significant deviations from the protocol?  **For partial yes, criteria 1-4, for yes, criteria 1-8** | Review question | N | N | N | N | N | N | N | N | N | N | N | N | N | Y | Y | N | Y | N |
|  | Search strategy | N | N | N | N | N | N | N | N | Y | Y | N | N | N | Y | Y | N | Y | N |
|  | Inclusion/exclusion criteria | N | N | N | N | N | N | N | N | Y | Y | N | N | N | Y | Y | Y | Y | N |
|  | ROB assessment | N | N | N | N | N | N | N | N | N | N | N | N | N | N | Y | N | Y | N |
|  | Protocol registered | N | N | N | N | Y | N | N | Y | N | N | N | N | N | Y | Y | N | Y | N |
|  | Meta-analysis plan (if appropriate) | NA | NA | N | N | NA | N | NA | NA | NA | N | NA | NA | NA | N | NA | Y | NA | NA |
|  | Causes of heterogeneity plan | NA | NA | N | N | NA | N | NA | NA | NA | N | NA | NA | NA | N | NA | N | NA | NA |
|  | Justification for protocol deviations | N | N | N | N | N | N | N | N | Y | N | N | N | N | N | N | N | Y | N |
|  | **Yes/Partial Yes/No** | **No** | **No** | **No** | **No** | **No** | **No** | **No** | **No** | **No** | **No** | **No** | **No** | **No** | **No** | **P-Yes** | **No** | **Yes** | **No** |
| 4. Did the review authors use a comprehensive literature search strategy?  **For partial yes, criteria 1-3, for yes, criteria 1-8** | Searched at least 2 databases (relevant to research question) | Y | Y | Y | Y | Y | Y | Y | Y | Y | Y | Y | Y | Y | Y | Y | Y | Y | Y |
|  | Provided key word and/or search strategy | Y | Y | Y | Y | Y | Y | Y | Y | Y | Y | Y | Y | Y | Y | Y | Y | Y | Y |
|  | Justified publication restrictions (e.g., language) | N | N | N | N | N | N | N | N | N | N | Y | N | N | N | N | N | N | N |
|  | Searched the reference lists / bibliographies of included studies | Y | Y | Y | N | Y | N | Y | Y | Y | Y | Y | Y | Y | Y | N | Y | Y | Y |
|  | Searched trial/study registries | N | N | N | N | N | N | N | N | Y | N | N | N | N | Y | N | Y | N | Y |
|  | Included/consulted content experts in the field | N | N | Y | Y | N | N | N | N | Y | N | N | N | N | N | N | N | N | N |
|  | Where relevant, searched for grey literature | N | Y | N | N | N | N | N | N | N | Y | N | N | N | Y | N | Y | N | N |
|  | Conducted search within 24 months of completion of the review | Y | Y | Y | Y | Y | Y | Y | Y | Y | Y | Y | Y | Y | Y | Y | Y | Y | Y |
|  | **Yes/Partial Yes /No** | **No** | **No** | **No** | **No** | **No** | **No** | **No** | **No** | **No** | **No** | **P-Yes** | **No** | **No** | **No** | **No** | **No** | **No** | **No** |
| 7. Did the review authors provide a list of excluded studies and justify the exclusions? | Provided a list of all potentially relevant studies that were read in full-text form but excluded from the review | N | N | Y | Y | N | N | N | N | N | N | Y | N | N | N | N | N | N | N |
|  | Justified the exclusion from the review of each potentially relevant study | N | N | Y | Y | N | N | N | N | N | N | Y | N | N | N | N | N | N | N |
|  | **Yes/Partial Yes/No** | **No** | **No** | **Yes** | **Yes** | **No** | **No** | **No** | **No** | **No** | **No** | **Yes** | **No** | **No** | **No** | **No** | **No** | **No** | **No** |
| 9. Did the review authors use a satisfactory technique for assessing the risk of bias (RoB) in individual studies that were included in the review? | Name | 8 questions (unspecified tool) | Cochrane Tool | Risk of Bias Table in Review Manager | Risk of Bias Table in Review Manager | GRADE | Critical Appraisal Skills Programme (CASP) RCT Checklist | None | Cochrane Tool | Cochrane Tool | Cochrane Tool | Cochrane Tool | None | None | Quality Assessment Tool for Quantitative Studies | Cochrane Tool | Quality Assessment Tool for Quantitative Studies | Crowe Critical Appraisal Tool | None |
| RCTs  – Risk of bias was assessed for…  **For partial yes, criteria 1 and 2, for yes criteria 1-4** | Unconcealed allocation | N | Y | Y | Y | ND | N | N | Y | Y | Y | Y | N | N | N | Y | N | N | N |
|  | Lack of blinding of patients and assessors when assessing outcomes (unnecessary for objective outcomes such as all-cause mortality) | Y | Y | Y | Y | ND | Y | N | Y | Y | Y | Y | N | N | Y | Y | Y | N | N |
|  | Allocation sequence that was not truly random, and | Y | Y | Y | Y | ND | Y | N | Y | Y | Y | Y | N | N | N | Y | N | Y | N |
|  | Selection of the reported result from among multiple measurements or analyses of a specified outcome | N | Y | Y | Y | ND | N | N | Y | Y | Y | Y | N | N | Y | Y | Y | N | N |
|  | **Yes/Partial Yes/No/ Includes only NRSI** | **No** | **Yes** | **Yes** | **Yes** | **No** | **No** | **No** | **Yes** | **Yes** | **Yes** | **Yes** | **No** | **No** | **No** | **Yes** | **No** | **No** | **No** |
| NRSI  - Risk of bias assessed from…  **For partial yes, criteria 1 and 2, for yes criteria 1-4** | From confounding | NA | N | NA | NA | ND | NA | N | NA | NA | NA | N | N | NA | NA | N | NA | Y | N |
|  | From selection bias | NA | N | NA | NA | ND | NA | N | NA | NA | NA | N | N | NA | NA | Y | NA | Y | N |
|  | Methods used to ascertain exposures and outcomes, and | NA | N | NA | NA | ND | NA | N | NA | NA | NA | N | N | NA | NA | N | NA | Y | N |
|  | Selection of the report result from among multiple measurements or analyses of a specified outcome | NA | N | NA | NA | ND | NA | N | NA | NA | NA | N | N | NA | NA | Y | NA | N | N |
|  | **Yes/Partial Yes/No/ Includes only RCT (NA)** | NA | **No** | NA | NA | **No** | NA | **No** | NA | NA | NA | **No** | **No** | NA | NA | **No** | NA | **No** | **No** |
| 11. If meta-analysis was performed, did the review authors use appropriate methods for statistical combination of results? | |  |  |  |  |  |  |  |  |  |  |  |  |  |  |  |  |  |  |
| RCTs  **For yes, criteria 1-3** | The authors justified combining the data in a meta-analysis | NA | NA | N | N | NA | N | NA | NA | NA | N | NA | NA | NA | N | NA | N | NA | NA |
|  | AND they used an appropriate weighted technique to combine study results and adjusted for heterogeneity if present. | NA | NA | Y | Y | NA | Y | NA | NA | NA | Y | NA | NA | NA | Y | NA | Y | NA | NA |
|  | AND investigated the causes of any heterogeneity | NA | NA | N | N | NA | N | NA | NA | NA | Y | NA | NA | NA | Y | NA | Y | NA | NA |
|  | **Yes/No/No meta-analysis conducted (NA)** | NA | NA | **No** | **No** | NA | **No** | NA | NA | NA | **No** | NA | NA | NA | **No** | NA | **No** | NA | NA |
| NRSI  F**or yes, criteria 1-3** | The authors justified combining the data in a meta-analysis | NA | NA | NA | NA | NA | NA | NA | NA | NA | NA | NA | NA | NA | NA | NA | NA | NA | NA |
|  | AND they used an appropriate weighted technique to combine study results and adjusted for heterogeneity if present. | NA | NA | NA | NA | NA | NA | NA | NA | NA | NA | NA | NA | NA | NA | NA | NA | NA | NA |
|  | AND they statistically combined effect estimates from NRSI that were adjusted for confounding, rather than combining raw data, or justified combining raw data when adjusted effect estimates were not available | NA | NA | NA | NA | NA | NA | NA | NA | NA | NA | NA | NA | NA | NA | NA | NA | NA | NA |
|  | AND they reported separate summary estimates for RCTs and NRSI separately when both were included in the review | NA | NA | NA | NA | NA | NA | NA | NA | NA | NA | NA | NA | NA | NA | NA | NA | NA | NA |
|  | **Yes/No/No meta-analysis conducted (NA)** | NA | NA | NA | NA | NA | NA | NA | NA | NA | NA | NA | NA | NA | NA | NA | NA | NA | NA |
| 13. Did the review authors account for RoB in individual studies when interpreting/ discussing the results of the review?  **For Yes, criteria 1 OR 2** | Included only low risk of bias RCTs | N | Y | N | N | Y | N | N | Y | N | N | N | N | N | N | Y | N | N | N |
|  | OR, if RCTs with moderate or high RoB or NRSI were included the review, provided a discussion of the likely impact of RoB on the results | N | N | Y | N | Y | Y | Y | NA | N | N | N | N | N | N | NA | N | N | N |
|  | **Yes/No** | **No** | **Yes** | **Yes** | **No** | **Yes** | **Yes** | **Yes** | **Yes** | **No** | **No** | **No** | **No** | **No** | **No** | **Yes** | **No** | **No** | **No** |
| 15. If they performed quantitative synthesis did the review authors carry out an adequate investigation of publication bias (small study bias) and discuss its likely impact on the results of the review | Performed graphical or statistical tests for publication bias and discussed the likelihood and magnitude of impact of publication bias | NA | NA | N | N | NA | Y | NA | NA | NA | Y | NA | NA | NA | Y | NA | Y | NA | NA |
|  | **Yes/No/No meta-analysis conducted (NA)** | **NA** | **NA** | **No** | **No** | **NA** | **Yes** | **NA** | **NA** | **NA** | **Yes** | **NA** | **NA** | **NA** | **Yes** | **NA** | **Yes** | **NA** | **NA** |
