# Supplementary material for: Effectiveness and implementation of interventions for health promotion in urgent and emergency care settings: an umbrella review
Source: BMC Emerg Med. 2023 Apr 6;23:41. doi: 10.1186/s12873-023-00798-7 (PMC10080902; doi:10.1186/s12873-023-00798-7)
Supplement: Supplementary file 5 — Additional file 5: Table A5. Screening tools used in intervention studies. [file 12873_2023_798_MOESM5_ESM.docx]

**Additional File 5**

**Table A5. Screening tools used in intervention studies**

| **First author (year)** | **Screening tools** |
| --- | --- |
| **Alcohol** |  |
| Barata et al., (2017) | Self-reported questionnaires and biomarkers were used for screening including: AUDIT, AUDIT-C, NIAAA (National Institute on Alcohol Abuse and Alcoholism Guide). CAGE, Paddington Alcohol Test (PAT), positive test for alcohol, and self-report of ingesting alcohol within six hours prior to injury. In adolescents: ADQ (Adolescent Drinking Questionnaire), ADI (Adolescent Drinking Instruments) to assess alcohol intake; Adolescent Health Behaviour Questionnaire and SMAST (Short Michigan Alcoholism Screening Test) to evaluate alcohol-related injuries. Eleven studies used biomarkers (blood, breath or saliva tests). Additional instruments used to evaluate negative consequences of drinking alcohol and readiness to change: Drinker’s Inventory of Lifetime Consequences (DrInC), Readiness to Change Contemplation Ladder adapted for an ED treatment-seeking population of injured drinkers. |
| Diestelkamp et al., (2016) | All studies required self-reported alcohol use within 6 h prior to hospitalisation or alcohol use having led to hospitalisation as the central inclusion criteria. One study additionally included individuals who screened 8 or higher on the Alcohol Use Disorders Identification Test (AUDIT) (23.7% of the total sample). Another study also included adolescents who presented to the ED following a drug-related event. |
| Elzerbi et al., (2015) | *Examples included::* Rapid Alcohol Problems Screen plus 3 questions; Paddington Alcohol Test (PAT; A 10-item questionnaire which asked about depression, tobacco, cholesterol, primary care physician, alcohol and drug use; Three questions were used to identify hazardous drinkers; Modified Single Alcohol Screening Questionnaire (MSASQ); FAST Alcohol Screening Test, or a modified version of the Paddington Alcohol Test (PAT; Alcohol questions were embedded in a larger health and lifestyle screening survey and Drinker Inventory of Consequences Short Inventory of Problems; 18-item health screen which included questions related to smoking, exercise, seatbelt use, and quantity and frequency alcohol questions; 17-item health screen which included; questions related to smoking, exercise, seatbelt use, and quantity and frequency alcohol questions; Blood alcohol concentration, gamma glutamyl transpeptidase level, and short Michigan Alcoholism Screening Test. |
| Elzerbi et al., (2017) | *Examples included:* AUDIT; Alcohol questions were embedded in a larger health and lifestyle screening Survey; a 10-item questionnaire that asked about depression, tobacco use, cholesterol, primary care physician, and alcohol and drug use Blood alcohol concentration; Self-reported drinking 6 h before injury; at-risk drinking as per NIAAA guidelines; Alcohol, Smoking and Substance Involvement Screening Test; 20-min interview protocol adapted from the one used in the TrEAT study; Rapid Alcohol Problems Screen; Paddington Alcohol Test (PAT); 18-item health screen that included questions related to smoking, exercise, seat belt use, and questions about quantity and frequency of alcohol consumption; 17-item health screen that included questions related to smoking, exercise, seat belt use, and quantity and frequency-related alcohol questions; Modified Single Alcohol Screening Questionnaire (MSASQ); FAST Alcohol Screening Test, or a modified version of the Paddington Alcohol Test and modified Readiness Ruler; Self-administered, computerized instrument on sex, age, race, ethnicity, and education level, plus AUDIT-C, TLFB, and RAPI; Alcohol-related questions on a touchscreen computer; National Alcohol Screening Day Primary Care Screening Form adapted for emergency medicine (includes CAGE); Drinker Inventory of Consequences and Readiness to Change Questionnaire |
| Kodadek et al., (2020) | No data provided. |
| Kohler & Hofmann (2015) | Studies screened for hazardous drinking in form of self-reported alcohol consumption, blood alcohol concentration, alcohol in saliva or breath, high-risk behaviour in conjunction with alcohol use, or a combination of these. Two studies used AUDIT; three studies measured BAC (Blood alcohol concentration); other studies used non-specified self-report tools. |
| Landy et al, (2016) | The most commonly used measure was the Alcohol Use Disorders Identification Test (AUDIT) or AUDIT-C. Other commonly used measures were the CAGE, the Timeline Follow-Back (TLFB), and the Paddington Alcohol Test (PAT). Some of the less commonly used measures were the Severity of Alcohol Dependence Questionnaire (SADQ), the Alcohol Problems Questionnaire, and blood alcohol concentration. |
| McGinnes et al., (2016) | All participants were screened for alcohol use by either questionnaire or laboratory testing. AUDIT score and return visits to the ED were each assessed in two studies. |
| Newton et al., (2013) | Adolescent Drinking Questionnaire (ADQ); AUDIT-C; Timeline Follow-Back Calendar (TLFB); Amidx; Alcohol Frequency/Quantity Index. |
| Schmidt et al., (2016) | *Examples included:* AUDIT, PAT, SAPAS, Alcohol-related negative consequences (Amidx); Impaired Driving Scale; AUDIT-C and negative consequences (DRInC); self-report binge drinking days; |
| Simioni et al., (2015) | To identify AUD patients, 1) three studies used criteria that meet the DSM-IV definitions for AUDs, 2) three studies used screening tests validated for identifying patients with heavy drinking in the EDs, and 3) one study included patients admitted for acute intoxication, which can be interpreted as a sign of a likely AUD. |
| Taggart et al., (2013) | *Examples included:* AUDIT; or alcohol positive on admission; treated in ED following alcohol-related event or presented to ED having drank alcohol in previous 12 months. Most studies used self-report measures to assess alcohol-related harm and alcohol related negative consequences, though some used objective data (Department of Motor Vehicle data). |
| Yuma-Guerrero et al., (2012) | *Examples included:* Adolescent Drinking Index; Adolescent Drinking Questionnaire; Adolescent Health Behaviour Questionnaire on alcohol-related problems; Young Adult Drinking and Driving Inventory; Alcohol frequency/quantity index; Alcohol misuse index; Single binge drinking question; AUDIT; Timeline follow-back; Rutgers Alcohol problem index; Adolescent injury checklist; Frequency of driving within an hour or drinking |
| **Smoking** |  |
| Lemhoefer et al., (2017) | No summary data provided for screening instruments used or biological validation. |
| Pelletier et al., (2014) | Self-reported and/or biomarkers were used to assess smoking cessation. No data provided for the self-report tools used. |
| Rabe et al., (2013) | No summary data provided for screening instruments used or biological validation. |
