# Supplementary material for: Effectiveness and implementation of interventions for health promotion in urgent and emergency care settings: an umbrella review
Source: BMC Emerg Med. 2023 Apr 6;23:41. doi: 10.1186/s12873-023-00798-7 (PMC10080902; doi:10.1186/s12873-023-00798-7)
Supplement: Supplementary file 6 — Additional file 6: Table A6. Participant characteristics, intervention design and content, and comparator/control conditions: alcohol interventions. [file 12873_2023_798_MOESM6_ESM.docx]

**Additional File 6**

**Table A6. Participant characteristics, intervention design and content, and comparator/control conditions: alcohol interventions**

| **Author (year)** | **Participant characteristics** | **Intervention** | **Comparator/control conditions** |
| --- | --- | --- | --- |
| Barata et al., (2017) | Participants were aged 12-70 years old; in most studies individuals were older than 18 years; six studies surveyed adolescents and young adults aged 13 and 21. | Most studies employed a face-to-face BI delivered by healthcare personnel (nurses, doctors, or social workers) who had received specialised training.  A few studies used booster sessions delivered after the initial BI.  Patients were screened in ED and referred to an alcohol health worker (AHW) for 30 minutes assessment and discussion about drinking behaviour (1 study). | Examples included:  Standard care; no intervention; screening only; written handout with advice about alcohol-related risks; health information pack, brochure or leaflet; booklet (tailored or standard messages); a verbal 5-minute minimal intervention; 1 month telephone follow-up; weekly reminder for 12 weeks to complete follow-up survey; graphic illustration of alcohol use; information on local resources |
| Diestelkamp et al., (2016) | All except one study showed similar patterns of gender distribution with a weighted mean of 60.2% of participants being male. | Interventions lasted between 30 and 60 minutes; one study delivered 2 additional sessions, 1 addressing adolescents and parents together and 1 addressing parents only; of the remaining 6 studies, 1 offered counselling to parents in addition to the BI for the patients; six studies tested the effectiveness of a brief motivational intervention (BMI).  *Booster Sessions***:**  Four studies included BIs with booster sessions delivered by telephone, online, or in person. Frequency and durations varied from one booster (no duration provided) to two 20–30 min telephone boosters including assessment and counselling to 3 weekly web-based boosters lasting 10 min each. | Minimal active control groups such as standard care, educational brochures or feedback only (5 studies); BI compared with an enhanced BI (2 studies): an individual-level intervention plus family intervention and an individual-level intervention plus computer-delivered exercises based on drinking motives |
| Elzerbi et al., (2015) | No data | *Examples:*  Brief advice interview (lasting from 10 minutes to 30 minutes); referral to counselling; tailored/generic booklet; booster telephone call | Screening only; assessment only; treatment as usual; evaluation only; or minimal intervention e.g., an information leaflet |
| Elzerbi et al., (2017) | No data | *Examples:*  Self-help booklets; tailored/ generic message booklet; brief advice/counselling (5-60 minutes) – including a mixture of feedback, reviewing alcohol guidelines, discussing motivation and goals; single sessions and multiple sessions; telephone BI; computer generated feedback booster telephone call; personalised mailed feedback; specialised action plans | Screening only; assessment only; treatment as usual; evaluation only; or minimal intervention e.g., an information leaflet |
| Kodadek et al., (2020) | No data | Screening, BI and referral to treatment (SBIRT) (9 studies) | Usual care (8 studies); no control group (3 studies) |
| Kohler & Hofmann (2015) | Age of participants ranged from 13 to 25 years; females 9.7% to 67.8%. | Interventions lasted from 5 to 45 min and were usually longer than 20 min, or had a median length of 37 min  MI interventions included a treatment similar to the control intervention plus MI  MI groups also received additional ‘booster’ phone calls after 10 days, or after 1 and 3 months that were not part of the control interventions (2 studies)  Two trials included booster phone calls | Standard care, included written information (e.g., alcohol-use risk handout, educational brochure), a contact list (e.g., community resources, adolescent treatment facilities), a phone follow-up, or personal feedback |
| Landy et al, (2016) | No data | *Examples:*  Combinations of: BIs (5-30 minutes); motivational interviewing; use of FRAMES; tailored or generic booklets; computer generated feedback; written information about behaviour change; patient /health information leaflet; personalised feedback given verbally or by text message; action plans; list of contacts; graphic illustrations of risks of weekly alcohol consumption; referral to treatment | *Examples:*  None; screening only, screening and additional information; a list of resources; referral list; 5 minutes advice; help for drinking when requested; questionnaire and referral. |
| McGinnes et al., (2016) | Age ranged from 14 years to 75 years; two studies included 18- to 25-year-olds; higher prevalence of males (up to 79%) | Ultra-BI with a face-to-face interaction of 10min or less (6 studies); computer intervention (3 studies); mobile phone intervention (2 studies); pamphlet only (2 studies). | *Examples:*  Standard care; booklet; brochure; phone numbers for community organisations; referral to community resources; handout; assessment with no feedback; short feedback |
| Newton et al., (2013) | 34%-67% male; age ranged from 12 to 21 years | A targeted approach (BIs for alcohol-positive youth) (4 studies); a universal approach (BIs for youth with a recent history of alcohol or other drug use) (5 studies); Seven studies included MI;  Interventions primarily delivered one-on-one (youth + intervention deliverer); 1 study included parental involvement; 1 study included computer delivery | Information handout and list of community resources (3 studies); a handout on community resources only (1 study); comparison of one-on-one MI (control group) to one-on-one MI, followed by a family-based MI ‘‘check-up’’ (intervention group) (1 study); brief (5 minutes) advice (termed standard care) to youth in the control group (1 study); standard medical care (3 studies) |
| Schmidt et al., (2016) | Participants aged 13 years or older; 5 studies included adolescents under 18 years; All studies included both genders, except for one investigating only men. | Face-to-face intervention (22 studies): included 'brief' BI (8 studies/9 publications) and extended BI (14 studies/18 publications)  Brief BI = 5-10 minutes (median 5) and included individual feedback with brief advice or a brief motivational interview; extended BI = 15-40 minutes (median 30), with a stronger focus on motivational elements  Booster sessions: One booster session 5-30 minutes duration (median 15) between 2 weeks and 3 months after discharge (8 studies),  Non-face-to-face interventions (6 studies): included interactive computer programme (1 study), printer computer-generated feedback (2 studies), leaflets (1 study), text messages (2 studies)  Most BI conducted after assessment, before patients discharged from ED; 7 studies BI scheduled for separate appointment | Four categories: treatment as usual or no intervention (15 publications); provision of general information leaflets (10 publications); specific intervention (7 publications) comprises either handout plus brief counsellor contact (e.g., unspecific, empathic advice), weekly text message reminders or personalized feedback; short form of BI (brief advice) (1 publication)  To investigate potential screening reactivity effects, five of the 28 studies additionally employed ‘not-assessed’ or minimally assessed control groups. |
| Simioni et al., (2015) | No data | Onsite extended BI (2 studies, CCTs); onsite brief advice (2 studies, 1RCT, 1NRCT); onsite brief advice with direct referral to a post-discharge single-session BI (1 study, RCT); a post-discharge letter without onsite intervention (1 study, RCT); referrals to a post-discharge two session BI and to an extended post-discharge intervention, without onsite intervention (1 study, RCT) | No active control conditions (four studies); active control conditions such as leaflets (three studies) |
| Taggart et al., (2013) | No data | MI (four studies); other forms of BIs (3 studies); interventions varied in type, length, who administered the intervention, and inclusion of booster sessions. | *Examples included*: standard care; weekly text messages; brochure in ED; assessments and brief handout; personalised feedback (1-3 mins) with a 5-10 min telephone booster at 1 and 3 months |
| Yuma-Guerrero et al., (2012) | Ages of patients varied; study with youngest age boundary included patients 12 to 20 years of age; study with highest age boundary included patients 18 to 24 years of age. | MI used as the foundation for the intervention (six out of seven studies); examples of additional intervention content: List of treatment resources; personalised feedback sheet; laptop-based interactive programme; worksheets; telephone booster at 10 days or 1 and 3 months; referral to community resources/ treatment | *Examples included:* handout on avoiding drinking and driving and a list of resources for treatment; standard medical care; standard care (defined as five minutes of brief advice and written resources); feedback only; screening survey only; additional assessments |

AHW = Alcohol Health Worker; AUD = Alcohol use disorder; BI = Brief intervention; COM-B = Capability, Opportunity, Motivation – Behaviour; ED = Emergency Department; RCTs = Randomised control trials; SBIRT = screening, brief intervention and referral to treatment; SMD = standardised mean differences
