# Supplementary material for: Effectiveness and implementation of interventions for health promotion in urgent and emergency care settings: an umbrella review
Source: BMC Emerg Med. 2023 Apr 6;23:41. doi: 10.1186/s12873-023-00798-7 (PMC10080902; doi:10.1186/s12873-023-00798-7)
Supplement: Supplementary file 7 — Additional file 7: Table A7. Participant characteristics, intervention design and content, and comparator/control conditions: smoking interventions. [file 12873_2023_798_MOESM7_ESM.docx]

**Additional File 7**

**Table A7. Participant characteristics, intervention design and content, and comparator/control conditions: smoking interventions**

| **Author (year)** | | **Participant characteristics** | **Intervention** | **Comparator/control conditions** |
| --- | --- | --- | --- | --- |
| Lemhoefer et al., (2017) | No data | | MI or cessation advice (4 studies); booster telephone calls (3 studies); study participants received Nicotine Replacement Therapy (NRT) free of charge (3 studies); heterogeneity in self-help materials, brochures and referrals to telephone quit lines. | *Examples included:* Personalised cessation advice and printed self-help materials; brochure and quit line information only; |
| Pelletier et al., (2014) | No data | | 6 broad categories of interventions: administration of self-help materials (pamphlets; faxed referrals to other programs, e.g., tobacco cessation phone programs); delivery of brief advice to quit; counselling; nicotine replacement therapy (NRT); MI-based interventions. | Most studies compared 2 or more interventions; no standard control group;  *Examples included:*  Self-help material e.g., single brochure/pamphlet, brochure with referral information or tobacco cessation phone line, very brief advice in clinic. |
| Rabe et al., (2013) | No data | | Variations in use of MI and referral to a smoking cessation programme at baseline; number and kind of additional interventions and use of booster phone calls differed across studies  Examples: Referral to smoking cessation programme, counselling or MI (up to 30 minutes), booster phone calls | *Examples included:* brief advice or brief counselling, self-help brochures/manual or pamphlet; referral sheet for local smoking cessation resources |

BI = Brief Intervention; ED = Emergency Department; ETC = emergency department-initiated tobacco control; MI = motivational interviewing; RR = relative risk; SMD = standardised mean difference
